# Effectiveness and Safety of Budesonide/Formoterol in Asthma: A Systematic Review

**DOI:** 10.3390/healthcare14131864

**Published:** 2026-06-26

**Authors:** Nam Xuan Vo, Huong Lai Pham, Han Tue Ho, Khoi Quoc Chung, Tien Thuy Bui

**Affiliations:** 1 Faculty of Pharmacy, Ton Duc Thang University, Ho Chi Minh City 700000, Vietnam; laihuong49@gmail.com (H.L.P.); hotuehan.st@tdtu.edu.vn (H.T.H.); chungquockhoi.st@tdtu.edu.vn (K.Q.C.); 2 Faculty of Pharmacy, Le Van Thinh Hospital, Ho Chi Minh City 700000, Vietnam; bttien.ths.tcqld23@ump.edu.vn

**Keywords:** efficacy, effectiveness, safety, severe exacerbation, budesonide/formoterol, asthma, maintenance and reliever therapy

## Abstract

**Background/Objectives:** Asthma’s preventable burden is heavily driven by severe exacerbations (SE). Replacing standalone short-acting β2-agonists (SABAs), which risk reducing patient tolerance, with inhaled corticosteroid/long-acting β2-agonist combinations optimizes care through maintenance, reliever, and maintenance-and-reliever therapy (MART). This systematic review and meta-analysis evaluated the efficacy, effectiveness, and safety of Budesonide/Formoterol (B/F). **Methods:** PubMed, Cochrane, and Embase were searched for randomized controlled trials (RCTs) and non-randomized controlled trials (non-RCTs) through May 15, 2026. Bias was assessed via the Cochrane Risk of Bias tool version 2 (RoB 2) and the Risk ff Bias in Non-randomized Studies of Interventions (ROBINS-I) version 2.0. Primary outcomes (time to first SE, annual SE rate) were pooled using a random-effects meta-analysis, yielding hazard ratios (HRs) and rate ratios (RRs). **Results:** We included 19 studies (15 RCTs, 4 non-RCTs) comprising 104,600 patients (primarily aged ≥12 years with mild-to-severe asthma). Most RCTs had a low risk of bias, whereas the non-RCTs had a high risk of bias. B/F MART significantly delayed the first SE and reduced annual rates versus Budesonide + SABA (HR = 0.57; RR = 0.55), B/F + SABA (HR = 0.62; RR = 0.58), and Fluticasone/Salmeterol + SABA (HR = 0.75; RR = 0.72). As-needed B/F reduced first SE hazard and annual rates versus SABA alone (HR = 0.43; RR = 0.42). Compared with Budesonide + SABA, it delayed the first SE (HR = 0.85) but showed non-significant rate reductions (RR = 0.90). Adverse events were balanced between groups over 12–52 weeks. **Conclusions:** B/F MART demonstrates high efficacy in mitigating the risk of the first SE. However, limited trial data leave the evidence for maintenance or reliever regimens controversial. Across all regimens, B/F is well-tolerated within 6 to 12 months.

## 1. Introduction

Asthma is a common chronic inflammatory airway disease characterized by variable respiratory symptoms, including wheezing, chest tightness, shortness of breath, and cough, as well as expiratory airflow changes [1]. According to the Global Burden of Disease 2019 data, asthma affected an estimated 262 million people globally across all age groups [2]. While childhood asthma has a high incidence and prevalence rate [2], the burden of asthma is increasingly shifting towards adolescents and adults. Many contemporary studies estimate that approximately 40–60% of adolescents and adults with asthma have uncontrolled disease [3,4,5,6]. Research on young adults (15–39 years old) shows that mortality and Disability-Adjusted Life Years increased from 1990 to 2019 [2], and the Global Burden of Disease 2021 findings indicate that this substantial health loss is concentrated in older age groups [7,8]. The burden of asthma in adolescents and adults is further compounded by the complexity of individual airway impairments [9,10] and comorbidities [11,12]. However, the most concerning burden of asthma remains acute exacerbations, with up to one in five patients experiencing a severe exacerbation (SE) or hospitalization each year, and over 8–12% of all patients have an exacerbation annually [13,14]. Furthermore, exacerbations are the leading cause of preventable death and frequent hospitalizations [15,16] and account for a large proportion of total medical costs [17,18]. In US adults with moderate or severe persistent asthma, patients with ≥ 1 exacerbation had nearly double the annual asthma-related costs ($1740 vs. $847) and significantly higher total costs ($9223 vs. $5011) compared to patients without exacerbations [18].

According to GINA, the primary goal in asthma management comprises not only the control of current symptoms but also the minimization of future risks (e.g., exacerbations, airflow limitation, and drug side effects) [1]. In clinical practice, however, achieving these goals has historically been impeded by traditional treatment paradigms. For many years, GINA guidelines recommended as-needed short-acting beta-agonists (SABAs) as the initial treatment for mild asthma (Step 1) and as the standard reliever alongside inhaled corticosteroid (ICS)-based maintenance therapy for more severe disease (Steps 2–5) [19]. This approach inadvertently created a patient misconception that rapid symptomatic relief equates to effective disease control. Consequently, patients often overuse SABAs while neglecting maintenance anti-inflammatory therapy [20,21], which increases the risk of disease progression and death. Across larger cohorts, the use of ≥3 SABA inhalers/year was associated with approximately a 1.5- to 2-fold higher risk of severe exacerbations and up to a 2- to 2.4-fold higher risk of death, compared to the use of ≤2 inhalers/year [22,23,24,25]. For that reason, treatment guidelines have undergone a major shift, prioritizing the initiation of ICS-containing therapies to target underlying inflammation and prevent exacerbations [26]. Crucially, evidence consistently demonstrates that adding a long-acting β2-agonist (LABA) to an ICS regimen provides superior improvements in lung function and symptom control compared to ICS alone [27,28]. In this context, Budesonide/Formoterol (B/F) occupies a central role in modern treatment strategies [29,30,31,32], leveraging the synergistic benefits of Budesonide’s anti-inflammatory action and Formoterol’s rapid, sustained bronchodilation [33]. This allows for the flexible use of B/F in both maintenance and reliever therapy (MART) and a fixed maintenance regimen [34].

Maintenance and reliever therapy (MART), often referred to as single maintenance and reliever therapy (SMART), is an asthma management strategy in which a single inhaler containing a combination of an inhaled corticosteroid (ICS) and the fast-acting, long-acting β2-agonist (LABA) Formoterol is used for both regular daily maintenance and as-needed relief of breakthrough symptoms [35,36,37,38]. This approach leverages the rapid onset of Formoterol—which provides symptom relief as quickly as short-acting β2-agonists (SABAs)—to ensure that every reliever actuation also delivers an anti-inflammatory dose, directly targeting underlying airway inflammation as symptoms arise [39,40,41]. Clinical evidence consistently demonstrates that MART significantly reduces the risk of severe exacerbations and provides equal or better asthma control with a lower total daily steroid load than conventional fixed-dose regimens [35,36]. However, while MART is approved in over 120 countries and is the preferred treatment track in the Global Initiative for Asthma (GINA) guidelines, the U.S. Food and Drug Administration (FDA) has not approved Budesonide/Formoterol for as-needed or MART usage [37,42]. This regulatory status in the United States is primarily due to the FDA’s rejection of data from the dry-powder inhaler (Turbuhaler) used in the majority of MART clinical trials, resulting in U.S. product labeling that remains restricted to maintenance indications only [37].

Modern asthma management has undergone a significant transformation through the application of personalized medicine and biologic therapies, specifically for patients with severe asthma who remain uncontrolled despite high-dose inhaled corticosteroids and secondary controllers [43]. This paradigm shift moves away from a traditional “one-size-fits-all” approach toward strategies tailored to specific inflammatory endotypes, primarily categorized as Type 2 (T2)-high or T2-low inflammation [44]. Biologic precision medicine has revolutionized the treatment landscape by offering monoclonal antibodies that target specific molecular drivers of airway inflammation [43,44]. These advanced therapies are now essential for individuals failing to respond to conventional maintenance treatments, aiming to eliminate the heavy burden of chronic symptoms and frequent, potentially life-threatening exacerbations [44,45]. By integrating clinical features with molecular biology, personalized medicine seeks to achieve optimal control and significantly improve the lives of those with the most severe forms of the disease [43].

Although the benefits of B/F are clear for adolescents and adults, several gaps remain that require further investigation. The evidence base of the B/F is dominated by RCTs, with most systematic reviews and meta-analyses focusing mainly on the efficacy outcomes of a specific B/F regimen, such as as-needed B/F versus maintenance ICS + SABA [46], MART versus fixed-dose ICS/LABA + SABA [47], or B/F versus other ICS/LABA [48]. Consequently, there is limited evidence regarding the effectiveness and safety of B/F in real-world clinical settings. By synthesizing evidence from both RCTs and non-RCTs, this systematic review aims to evaluate the efficacy, effectiveness, and safety of B/F regimens (e.g., reliever, maintenance, and MART) over at least 12 weeks in adolescents and adults, thereby identifying the benefits of B/F in a clinical context versus real-world conditions to support future asthma management guidelines.

## 2. Materials and Methods

### 2.1. Searching Strategy

This systematic review was conducted following the guidance of the Preferred Reporting Items for Systematic Reviews and Meta-Analyses 2020 (PRISMA 2020) guidelines [49]. The protocol was registered in the Open Science Framework (OSF): https://osf.io/w8r9g (accessed on 9 April 2026).

We used three electronic databases, including PubMed, Cochrane, and Embase, to identify relevant trials from database inception until 15 May 2026. The preliminary search was constructed based on the PICO criteria: (P) asthmatic patients, covering adolescents and adults; (I) Budesonide/Formoterol metered-dose inhaler; (C) other asthma regimens; and (O) time to first severe exacerbation for RCTs, or exacerbation outcomes for non-RCT studies.

Based on these primary criteria, related topic keywords were selected and used as search terms in the search bar, including “asthma”, “budesonide”, “formoterol”, “exacerbation”, “efficacy”, or “safety”. Multiple search attempts were conducted to identify the most appropriate terms and ensure sufficient coverage of relevant trials, as listed in Table S1 (supplemental data). The full search strategy covering all publications is as follows: (Asthma) AND ((Budesonide) AND ((Formoterol) OR (Formoterol fumarate))) AND ((Efficacy) OR (Effectiveness) OR (Safety)).

### 2.2. Inclusion and Exclusion Criteria

Trials were qualified if they met the following inclusion criteria: (1) the study design fell into the RCT (for efficacy assessment) or non-RCT (for effectiveness evaluation, such as retrospective cohort or other observational studies) category; (2) the trials reported at least one intervention arm using a fixed-dose B/F combination, with a clearly specified regimen (MART, maintenance, or reliever use); (3) the comparators belonged to an approved asthma regimen with a clear regimen type (for instance, ICS/LABA as maintenance therapy, a SABA as-needed strategy, etc.) and the specific active ingredients of the comparator were clearly stated; (4) the patients were diagnosed with asthma, including adolescents and adults (preferably ≥12 years of age), and were at risk of developing exacerbations; (5) the outcomes focused on exacerbation endpoints, including the time to the first SE for RCTs, or SE outcomes for real-world/non-RCT studies, with a clear endpoint assessment/definition; and (6) full-text access was available, and papers were written in English.

In contrast, trials were excluded if they met any of the following conditions: (1) studies were out of the RCT/non-RCT scope or beyond the efficacy/effectiveness question (e.g., cross-over studies, pharmacokinetic studies, trial-based cost-effectiveness analyses, etc.); (2) B/F was not used as a fixed-dose combination inhaler, including use in separate devices, titrated doses, or used only as an add-on component in triple therapy; (3) the comparators were B/F at different doses only; (4) the patients had asthma with major comorbidities such as COPD, or the study population was restricted to children only; (5) the outcomes did not report sufficient statistical results for the time to the first SE in RCTs or SE in non-RCT studies (e.g., effect estimates were not available); or (6) the full text could not be retrieved and/or the publication language was not English.

### 2.3. Data Extraction

The following information was extracted after retrieving the full text: the first author, year of publication, study design, trial ID/registry number, participants’ age, asthma severity, baseline forced expiratory volume in one second (FEV1%), B/F indication/regimen, comparator regimen, dose, sample size for the efficacy analysis, study setting (place of conduct), and sponsor/funding.

Our primary outcomes focus on exacerbation efficacy/effectiveness. For RCTs, the primary efficacy outcome is the time to the first severe exacerbation (reported as a hazard ratio [HR], when available). Time to the first SE is defined as a deterioration in asthma symptoms that necessitates a hospitalization/emergency department visit, or the use of oral corticosteroids (OCS) for at least 3 days [50,51].

The number of patients experiencing at least one severe exacerbation, the total number of severe exacerbation events, and the annual severe exacerbation rate were also collected as supporting efficacy data for Budesonide/Formoterol. The annual severe exacerbation rate is expressed as events per patient-year and reported as a rate ratio (RR) or hazard ratio (HR), depending on the study reporting. Regarding non-RCT studies, because the time to the first severe exacerbation is often unavailable in real-world studies and the definition of a severe exacerbation is not fully standardized across studies, the exacerbation rate was used to assess Budesonide/Formoterol effectiveness. Secondary outcomes focus on safety, including the incidence of adverse events and the most common symptoms reported. Serious adverse events and the discontinuation rate were also collected.

### 2.4. Data Synthesis

The research applied both a meta-analysis and a narrative synthesis. First, the included studies were classified into an RCT-based synthesis and an observational trial-based synthesis. Then, the studies were grouped by Budesonide/Formoterol (B/F) clinical use, whether as a MART, maintenance, or reliever regimen. Within each B/F indication, comparators were further stratified by pharmacological regimen type, such as ICS/LABA + SABA, ICS-only, or ICS + SABA. This helped distinguish the magnitude of the B/F benefit in controlled versus real-world settings. 

For efficacy (RCTs), the time to the first SE and annual SE rates were meta-analyzed using diamond forest plots based on comparator subgroups within each B/F regimen. In studies that did not report 95% CIs or sufficient compatible data for pooling, the results were displayed in tables, reporting HRs/RRs and *p*-values where available.

As severe exacerbation reduction is the central measure to assess Budesonide/Formoterol efficacy, in cohort studies, where time-to-event estimates are often unavailable, the exacerbation rate (events/patient/year) was used as the primary measure of effectiveness. Additionally, exacerbation-related hospitalizations were summarized as supportive evidence. Moreover, the overall clinical status of patients was evaluated based on the mean baseline values and the mean changes in FEV1 and ACQ-5/ACT. SDs were included where available. In terms of safety, the incidence and the most common adverse events were noted and compared across trials to assess consistency. The prevalence of serious adverse events and the discontinuation rate due to AEs were summarized to determine the tolerability of B/F and the comparators. All numerical values were presented in summary tables for visual comparison.

### 2.5. Statistical Analysis

A meta-analysis was performed when at least two studies per subgroup reported HRs for the time to the first severe exacerbation or rate ratios/RRs for the annual severe exacerbation rate, along with the corresponding 95% CIs. For studies reporting only a percentage risk reduction with 95% CI ranges, the values were converted to rate ratios to allow their inclusion in the meta-analysis where appropriate. For both outcomes, the input HRs/RRs were log-transformed, and standard errors (SEs) were calculated from the reported 95% CIs, from which the variances were obtained by squaring the SEs. A random-effects model using the DerSimonian and Laird method was applied to calculate all pooled estimates to account for potential between-study variance. All pooled results were presented as HRs or RRs with 95% CIs using Microsoft Excel.

Statistical heterogeneity among the trials was quantified using Cochran’s Q and I^2^ statistics. All statistical tests were two-sided, with a *p*-value < 0.05 considered statistically significant.

### 2.6. Risk of Bias Evaluation

#### 2.6.1. For RCTs

To ensure a comprehensive methodological assessment of the RCTs, we used the Cochrane Risk of Bias tool version 2 (RoB 2) [52]. We selected RoB 2 over the previous version (RoB 2) [53] because it provides a more detailed evaluation through domain-specific signaling questions. The RoB 2 checklist comprises five domains to assess different sources of bias in included randomized controlled trials. Each domain is judged at one of three levels of risk of bias: low risk of bias, some concerns, and high risk of bias. To conclude each domain judgment, the researchers answer the signaling questions within each domain and then apply the prespecified algorithm provided by the RoB 2 tool. The overall risk-of-bias judgment for each study outcome is determined using the RoB 2 algorithm and is primarily driven by the highest level of bias identified across the five domains.

#### 2.6.2. For Non-RCTs

The risk of bias in the three included non-randomized studies (non-RCTs) was evaluated using the ROBINS-I tool (Risk Of Bias In Non-randomized Studies of Interventions), version 2.0 (2024 update) [54]. This tool assesses bias across seven domains. The domains were rated on a four-point scale ranging from low to high risk of bias: low, moderate, serious, and critical. Similar to the risk-of-bias assessment for the RCTs, the domain-level judgments were graded using the ROBINS-I algorithm for each outcome. The overall methodological risk-of-bias judgment for that outcome was determined by the worst (highest) judgment among the seven domains. Notably, for Domain 1 (bias due to confounding), we applied Variant A (baseline confounding). This approach was appropriate for the observational nature of the included studies, which compared treatment effectiveness based on initial group assignment, where key confounders were present before the intervention started. The assessment was performed independently by two reviewers. Any disagreement was resolved through discussion or by a third reviewer.

## 3. Results

### 3.1. Selecting Studies

**Figure 1:**
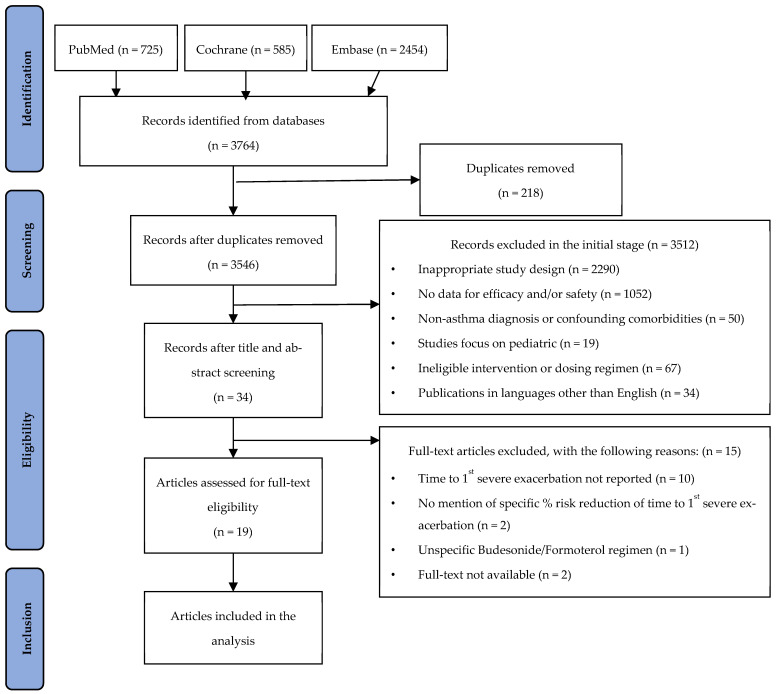
PRISMA flow diagram for identifying and selecting articles.

A comprehensive search across the relevant databases identified 3764 studies, including 725 from PubMed, 585 from Cochrane, and 2454 from Embase (Figure 1). After removing 218 duplicate records, 3764 publications remained for title and abstract screening. Of these, 3546 studies were excluded for failing to meet the selection and exclusion criteria. Consequently, the remaining 34 studies underwent further full-text review. Ten RCTs mentioning asthma exacerbation were initially prioritized as potentially relevant during the title and abstract screening, suggesting that they might report the primary outcome of interest (e.g., the time to the first severe exacerbation). However, the subsequent full-text assessment revealed that none provided specific data on the time to the first severe exacerbation. Among these ten, six RCTs focused solely on the exacerbation rate [55,56,57,58,59,60], while the remaining four assessed the time to the first mild [61,62] or mixed severities [63], or recorded no severe events [64]. Two other RCTs reported only the *p*-value for the time to the first severe exacerbation, without providing hazard ratios or the percentage reduction in the risk of an SE [65,66]. Two articles were not accessible in full text [67,68]. The remaining article was excluded because the B/F regimen (e.g., fixed-dose, MART) could not be determined due to limitations in database coding [69]. Ultimately, 19 articles that fully met the criteria were included in the final dataset.

### 3.2. Characteristics of Included Trials

The overall features of the eligible trials are summarized in Table 1. A total of 19 studies met the inclusion criteria, comprising 15 RCTs [35,39,40,50,51,70,71,72,73,74,75,76,77,78,79] and four non-RCT studies [41,80,81,82]. The RCTs generally evaluated both the efficacy and safety of Budesonide/Formoterol (B/F), whereas the real-world trials focused primarily on comparative effectiveness.

The majority of the included trials (17/19) restricted inclusion to adolescents and adults aged 12 years and older [35,39,40,41,50,51,70,71,72,74,75,77,78,79,80,81,82]. One study did not explicitly report the age range of its participants [80]. While O’Byrne et al. (2005) was the only trial to include children aged 4–11 years, this subgroup accounted for only 12% of the total cohort (341/2760 patients) [73]. Furthermore, the study design employed a pre-specified stratification ratio of 8:1 for adults to children [73]. Consequently, the pooled results remain predominantly representative of the adult and adolescent population [73]. Regarding asthma severity, 13/19 studies (68.4%) enrolled patients with moderate-to-severe persistent asthma, mostly in trials evaluating B/F as maintenance and reliever therapy (MART) or as maintenance treatment [35,39,40,41,50,72,73,76,77,78,80,81,82]. The remaining six trials (31.6%) focused on mild-to-moderate asthma and predominantly assessed B/F as an as-needed reliever [51,70,71,79], with a smaller number examining B/F as maintenance [75] or as the MART regimen [74]. In addition, the baseline lung function tended to be better in the mild-to-moderate group, with the mean %FEV_1_ generally between 75% and 89% predicted [51,70,71,74,75,79]. Trials enrolling patients with moderate-to-severe disease showed a wider range of airflow limitation, with the mean baseline FEV_1_ typically between 41% and 91.6% [35,39,40,41,50,72,73,76,78,81].

With respect to treatment strategies, B/F used as MART was the most common approach, reported in 11/19 studies. The remaining studies used B/F either as an as-needed reliever (4/19 studies, 21.05%) [51,70,71,79], or as maintenance therapy (4/19 studies, 21.05%) [75,77,81,82]. Among MARTs, the most frequent comparators were fixed-dose ICS/LABA maintenance + SABA as-needed (7/11 MART studies, 63.6% [35,39,40,50,73,76,78]), whereas a smaller number compared B/F to ICS maintenance + SABA as-needed [72,73,74]. In the reliever trials, B/F was compared either with SABA-only regimens or ICS + SABA [51,70,71,79]. For the maintenance-only studies, B/F was compared with other ICS/LABA combinations [81,82] or with Budesonide monotherapy (ICS-only regimen) [75].

Across all included trials, the B/F formulations were limited to 80/4.5 µg, 160/4.5 µg, and 200/6 µg per inhalation. Among these, B/F 160/4.5 μg was the most frequently used strength, reported in 6/19 studies (31.6%) [35,39,40,50,72,78]. Sample sizes ranged from over 100 to over 50,000 patients.

The efficacy of Budesonide/Formoterol (B/F) demonstrates a nuanced relationship with dosage and regimen across various studies. Maintenance and reliever therapy (MART) has consistently shown superior efficacy in reducing severe exacerbations compared to higher-dose fixed-maintenance regimens, such as Budesonide alone or Fluticasone/Salmeterol [39,50,74,78]. Crucially, the sources indicate that this superior control is often achieved with a significantly lower total daily dose of inhaled corticosteroids (ICS) than traditional fixed-dose strategies [39,46,50,72,74,78]. While higher ICS doses are generally associated with greater improvements in lung function and reductions in airway inflammation, the timing of administration in response to symptoms (as seen in MART) appears to be a more critical factor for preventing exacerbations than simply increasing the fixed maintenance dose [46,73,79]. The heterogeneity in treatment protocols across studies—ranging from low-dose maintenance (80/4.5 µg) to medium-dose (160/4.5 µg) twice daily—reflects the diverse baseline clinical needs and asthma severities of the studied populations [35,73,74,75,78].

Regarding safety, B/F is generally well-tolerated at both low and medium doses, with adverse event (AE) profiles comparable to those of short-acting beta-agonists or fixed-dose ICS/LABA combinations [39,50]. However, the sources highlight a potential dose-dependent increase in side effects, particularly with long-term high-dose ICS exposure [71]. Systemic side effects, such as cortisol suppression, are more frequently observed at higher doses (e.g., >800 µg/day), whereas lower doses (200–400 µg/day) maintain a higher therapeutic index with minimal systemic activity [71]. Local adverse effects, including oropharyngeal candidiasis and dysphonia, also show a dose–response relationship, although their overall incidence remains low (ranging from 0.5% to 2.0%) in many trials [71,74,78]. Despite the variability in study designs and dosages, the collective evidence suggests that B/F-based strategies effectively balance efficacy and risk when tailored to individual patient needs and asthma severity [50,74,78,81].

In terms of sponsorship, most trials were sponsored by pharmaceutical companies, with AstraZeneca being the predominant funder (15/19 articles, 78.95%) [35,39,40,50,51,70,71,72,73,74,75,77,78,80,82]. Additional industry sponsors included GSK [75,81], and Merck [75]. Public and academic funding bodies were also represented, such as the Health Research Council of New Zealand [51,76,79]. Only one study did not specify any information related to funding sources [41].

### 3.3. Risk of Bias Assessment

#### 3.3.1. RoB 2.0 Tool for Evaluating RCT

The risk-of-bias assessment following the RoB 2.0 criteria is presented in Figure S1 [83]. In general, 12/15 (80%) trials were assessed as a low risk of bias [35,40,50,70,71,72,73,74,76,77,78,79]. The remaining studies did not meet the criteria for a low risk of bias, mainly due to concerns in Domain 4, “Bias in measurement of the outcome.” [39,51]. Fewer concerns were identified in Domain 2 [39] and Domain 5 [35]. As a result, three studies (16.5%) were judged as having a moderate risk of bias [39,51,75].

For the studies with an overall judgment of a moderate risk of bias, the choice of an open-label design (Domain 2) and the possibility that outcome assessors may have influenced the efficacy outcomes (Domain 4) contributed to increased concerns regarding overall study validity [39,51]. In another trial, although the study complied with several methodological standards, insufficient information about a prespecified protocol led to a moderate-risk judgment in Domain 5 [75].

#### 3.3.2. ROBINS-I Tool for Evaluating Non-RCT

The methodological quality assessment of the included non-randomized controlled trials (non-RCTs) is visually summarized as a traffic-light plot in Figure S2 [83]. In general, all reported non-RCTs were judged to have a serious risk of bias [41,80,81,82]. Most trials published in recent years (¾ articles) failed to adequately control baseline confounding (e.g., asthma severity and exacerbation history), leading to an overall judgment of Domain 1 as Serious [41,80,81]. In Tunceli et al.’s research, the overall serious judgment was mainly driven by selection bias because switchers were excluded from the efficacy analysis during follow-up. Consequently, Domain 3 was graded as Serious [82].

### 3.4. Efficacy

#### 3.4.1. Maintenance and Reliever Therapy (MART)

**Figure 2:**
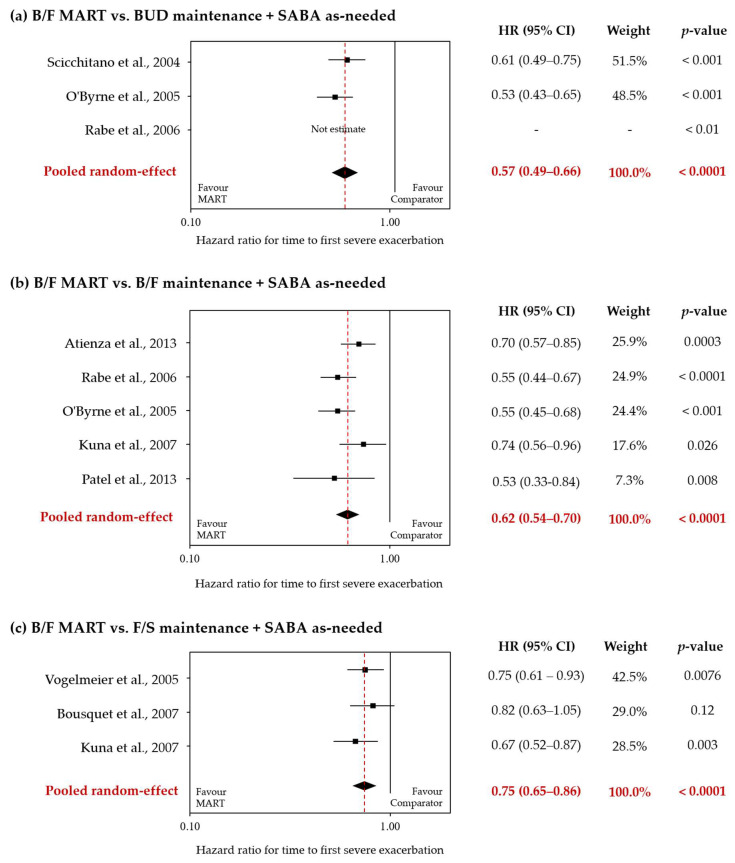
Forest plots displaying the hazard ratio (HR) for the time to first severe exacerbation in patients receiving Budesonide/Formoterol (B/F) MART compared with alternative therapies. The meta-analyses are stratified by comparator regimens: (**a**) B/F MART versus Budesonide (BUD) maintenance plus SABA as-needed [72,73,74]; (**b**) B/F MART versus B/F maintenance plus SABA as-needed [35,40,73,76,78]; and (**c**) B/F MART versus Fluticasone/Salmeterol (F/S) maintenance plus SABA as-needed [35,39,50]. Abbreviations: B/F = Budesonide/Formoterol, F/S = Fluticasone/Salmeterol, BUD = Budesonide, MART = maintenance and reliever therapy, SABA = short-acting β2-agonist, CI = confidence interval.

The efficacy of B/F as MART regarding the time to the first severe exacerbation is illustrated in Figure 2. Overall, B/F as MART significantly prolonged the time to the first severe exacerbation across all comparators, including the ICS/LABA maintenance + SABA as-needed regimen and the ICS maintenance + SABA as-needed subgroups. The greatest reduction was observed in the subgroup comparing B/F MART with Budesonide maintenance plus SABA as-needed, where B/F MART reduced the hazard of the first severe exacerbation by 43% (HR = 0.57, 95% CI 0.49–0.66, *p* < 0.0001, I^2^ = 0.0%) (Figure 2a).

In comparison with the ICS/LABA maintenance + SABA as-needed regimen, B/F MART was associated with a 38% reduction in the hazard of the first severe exacerbation compared with B/F maintenance plus SABA as-needed (pooled HR = 0.62, 95% CI 0.54–0.70, *p* < 0.0001, I^2^ = 33.6%) (Figure 2b). The efficacy of B/F MART was lower in the subgroup compared with Fluticasone/Salmeterol maintenance + SABA as-needed, although B/F MART still significantly reduced the hazard of the first severe exacerbation by 25% (pooled HR = 0.75, 95% CI 0.65–0.86, *p* < 0.0001, I^2^ = 0.0%) (Figure 2c).

**Figure 3:**
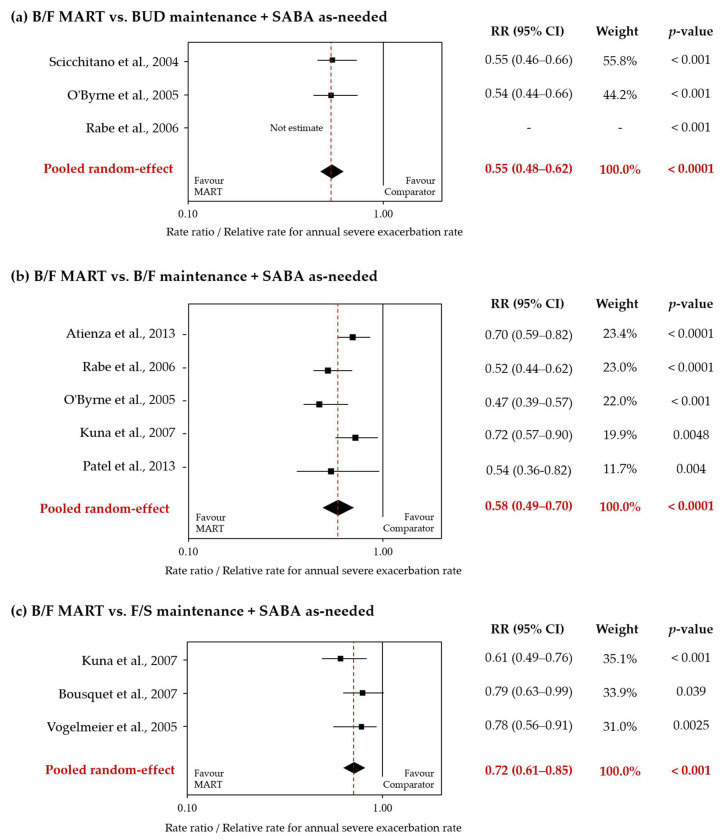
Forest plots displaying the risk ratio (RR) for annual severe exacerbations in patients receiving Budesonide/Formoterol (B/F) MART compared with alternative therapies. The meta-analyses are stratified by comparator regimens: (**a**) B/F MART versus Budesonide (BUD) maintenance plus SABA as-needed [72,73,74]; (**b**) B/F MART versus B/F maintenance plus SABA as-needed [35,40,73,76,78]; and (**c**) B/F MART versus Fluticasone/Salmeterol (F/S) maintenance plus SABA as-needed [35,39,50]. Abbreviations: B/F = Budesonide/Formoterol, F/S = Fluticasone/Salmeterol, BUD = Budesonide, MART = maintenance and reliever therapy, SABA = short-acting β2-agonist, CI = confidence interval.

The pooled estimate of B/F as MART for the annual severe exacerbation rate is illustrated in Figure 3. Regarding the annual severe exacerbation rate, B/F MART generally resulted in fewer severe exacerbations than the comparator regimens included, namely ICS/LABA maintenance + SABA as-needed [35,39,40,50,73,76,78] and ICS maintenance + SABA as-needed [72,73,74]. The greatest reduction was recorded in the subgroup comparing B/F MART with Budesonide maintenance + SABA as-needed, where B/F MART reduced the annual severe exacerbation rate by 45% (pooled RR = 0.55 (95% CI 0.48–0.62), *p* < 0.0001, I^2^ = 0.0%) (Figure 3c).

Compared with the strategy using Budesonide/Formoterol maintenance + SABA as-needed, B/F MART demonstrated a 42% decrease in annual severe exacerbation rate (pooled RR = 0.58, 95% CI 0.49–0.70, *p* < 0.0001, I^2^ = 73.0%) (Figure 3b). When the comparator was changed to Fluticasone/Salmeterol maintenance plus SABA as-needed, B/F MART showed a 28% reduction in annual severe exacerbation rate (pooled RR = 0.72, 95% CI 0.61–0.85, *p* < 0.0001, I^2^ = 38.5%) (Figure 3a). Similarly, the number of patients experiencing severe exacerbations and the total number of severe exacerbation episodes were consistently lower in the B/F MART groups across the three comparator subgroups (Table S2).

#### 3.4.2. Reliever Monotherapy

**Figure 4:**
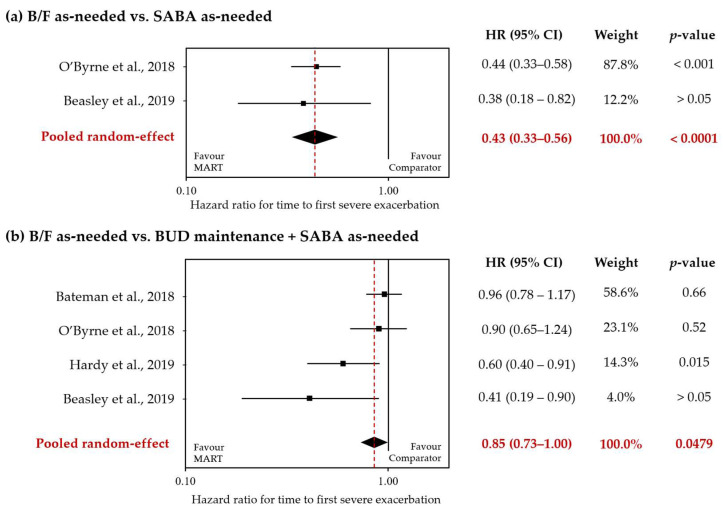
Forest plots displaying the hazard ratio (HR) for the time to first severe exacerbation in patients receiving Budesonide/Formoterol (B/F) as-needed therapy compared with alternative therapies. The meta-analyses are stratified by comparator regimens: (**a**) B/F as-needed versus SABA as-needed [51,70]; and (**b**) B/F as-needed versus Budesonide (BUD) maintenance plus SABA as-needed [51,70,71,79]. Abbreviations: B/F = Budesonide/Formoterol, BUD = Budesonide, SABA = short-acting β2-agonist, CI = confidence interval.

The efficacy of B/F as a reliever in prolonging the time to the first exacerbation is summarized in Figure 4. Patients using B/F as-needed had a 57% lower hazard of experiencing the first severe exacerbation compared with SABA as-needed during the 52-week treatment period (pooled HR = 0.43, 95% CI 0.33–0.56, *p* < 0.0001, I^2^ = 0.0%) (Figure 4a). In the subgroup compared with ICS maintenance plus SABA as-needed, B/F as-needed was associated with a 15% lower hazard of the first severe exacerbation after 52 weeks, with borderline statistical significance (pooled HR = 0.85, 95% CI 0.73–1.00, *p* = 0.0479, I^2^ = 60.7%) (Figure 4b).

**Figure 5:**
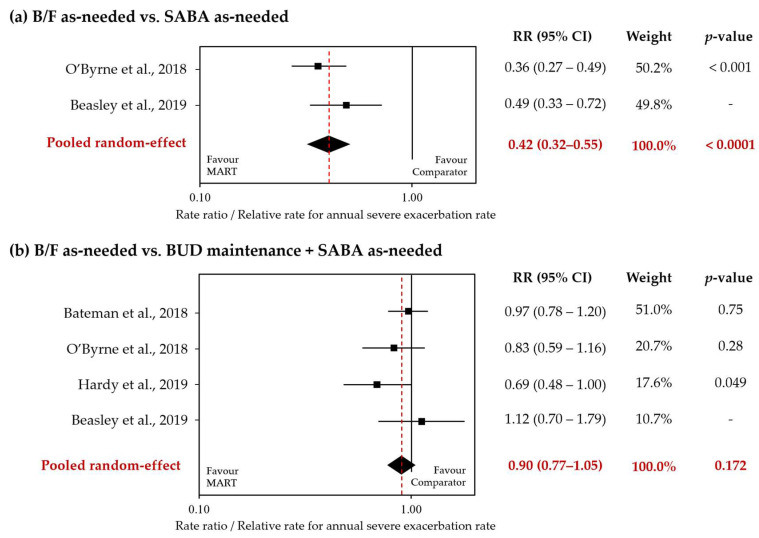
Forest plots displaying the risk ratio (RR) for annual severe exacerbations in patients receiving Budesonide/Formoterol (B/F) MART compared with alternative therapies. The meta-analyses are stratified by comparator regimens: (**a**) B/F as-needed versus SABA as-needed [51,70]; and (**b**) B/F as-needed versus Budesonide (BUD) maintenance plus SABA as-needed [51,70,71,79]. Abbreviations: B/F = Budesonide/Formoterol, BUD = Budesonide, SABA = short-acting β2-agonist, CI = confidence interval.

Moreover, the pooled estimate of the annual severe exacerbation rate for B/F as-needed, compared with other regimens, is summarized in Figure 5. In the subgroup compared with the SABA-only reliever, B/F as-needed was associated with a statistically significant 58% lower annual SE rate (Pooled RR = 0.42, 95% CI 0.32–0.55, *p* < 0.0001, I^2^ = 34.0%) (Figure 5a). However, the pooled effect of B/F as-needed compared with the ICS maintenance + SABA arms did not reach significance (pooled ratio = 0.90, 95% CI 0.77–1.05, *p* = 0.172, I^2^ = 15.1%) (Figure 5b). Across the two subgroups, fewer patients had at least one SE episode, and fewer SE cases were reported in the B/F as-needed regimen (Table S3).

#### 3.4.3. Maintenance Monotherapy

The efficacy of B/F as maintenance therapy is displayed in Table S4. In comparison with the ICS maintenance-only regimen, only one of the two trials reported a statistically significant reduction in the time to the first severe exacerbation. Peters et al. showed that B/F maintenance was associated with a 16% lower hazard of the first severe exacerbation during 26 weeks of treatment [77], whereas the 12-week trial by Lalloo et al. did not reach statistical significance [75]. Peters et al. also reported fewer patients experiencing at least one severe exacerbation and fewer total severe exacerbation events in the B/F maintenance group compared with the ICS maintenance-only group [77].

### 3.5. Effectiveness

The effectiveness of B/F as MART and maintenance therapy in a real-world studies is illustrated in Table 2. Overall, the exacerbation outcomes were mostly comparable between B/F and the comparators: no statistically significant differences were reported in 3/4 studies [41,80,81] while one study showed a significantly lower exacerbation rate with B/F compared with FP/SAL (*p* = 0.0255) [82].

The B/F MART regimen showed no significant difference in the severe exacerbation rate or the hospitalization rate due to exacerbations compared with ICS/LABA + SABA [80]. Huang et al. similarly found no significant difference in the mean change in annual acute exacerbations between B/F MART and FF/VIL MART [41]. Moreover, B/F maintenance provided a comparable exacerbation rate to the ICS/LABA option (Fluticasone/Salmeterol) over a 12-month follow-up [74,75], with ½ of the trial results being not statistically different [81]. Taken together, these real-world data suggest that B/F, as MART or maintenance, provides comparable effectiveness in reducing exacerbations.

Regarding lung function, the changes in FEV1 were small and not significantly different between the groups in both MART cohorts [41,80]. Asthma symptom control varied between the studies: Cheng et al. observed a statistically greater improvement in ACQ-5 with B/F than with F/S (*p* = 0.027), although the between-group difference did not reach the minimal clinically important difference (MCID) [80], while Huang et al. reported a larger increase in the ACT score in the FF/VIL MART group than in the B/F MART group (*p* < 0.001) [41].

### 3.6. Safety

#### 3.6.1. Maintenance and Reliever Therapy (MART)

The most common adverse event of Budesonide/Formoterol as MART are demonstrated in Table 3. Overall, B/F, used as a MART strategy at doses of 80/4.5 μg and 160/4.5 μg, is well tolerated over a 6- to 12-month treatment period. The incidence of side effects between B/F and comparators was comparable across trials, ranging from 38% to 61.4% for B/F and from 40% to 57.5% for ICS/LABA maintenance + SABA as-needed [39,64,65,68] and ICS maintenance + SABA as-needed [72,73,74]. The most common AEs were mild-to-moderate and focused on respiratory infections, including upper respiratory tract infections, nasopharyngitis, bronchitis, pharyngitis [35,40,72,74,76]. Less frequently reported AEs were candidiasis and hoarseness, which were noted in Rabe et al.’s research [78].

Serious adverse events were less than 8.2% and comparable between B/F and the comparators [35,39,40,50,73,76,78]. Similarly, the discontinuation rate due to AE was relatively low, as observed in trials evaluating B/F MART vs. ICS/LABA + SABA [76,78] and B/F maintenance vs. ICS + SABA [72].

#### 3.6.2. Reliever or Maintenance Monotherapy

Detailed data related to the adverse events of B/F for either reliever or maintenance purposes is shown in Table 4. Similar to MART, B/F used for relief purposes is well-tolerated, as it has an AE rate comparable to those of the SABA option and ICS maintenance + SABA as-needed therapy. The incidence varied among studies, ranging from 38% to 88% for B/F and 39.9% to 83.7% for the comparators [51,70,71,79].

The most common side effects of B/F (for reliever purposes or maintenance purposes) and the comparators are upper respiratory tract infection, nasopharyngitis, and asthma [51,70,71,79]. The discontinuation rate due to AEs was low (less than 3%) and not significant between the intervention groups [70,71,77]. In addition, the prevalence of SAEs is also comparable between B/F and the comparators in the majority of trials [51,70,77]. Notably, only the study by Beasley et al. [51] recorded a double SAE rate in the B/F relief group compared to Albuterol as-needed over 52 weeks. [51].

## 4. Discussion

### 4.1. Efficacy

Our findings reveal that using B/F as MART prolongs the time to the first SE and reduces the annual SE rate more effectively than regimens involving SABA as a reliever, such as ICS/LABA maintenance + SABA as-needed or ICS maintenance + SABA as-needed. The reduction in the risk of first SE with B/F MART was most pronounced compared with ICS maintenance + SABA as-needed, at 43%. In a previous systematic review, Jenkin et al. estimated around a 36–40% reduction in SE compared to high-dose ICS/LABA + SABA in patients treated with GINA step 3–4 [36], which is broadly aligned with our findings. Similarly, the network meta-analysis of RCTs by Rogliani et al. also reported a 46% reduction in SE risk in the B/F MART groups among individuals with moderate-to-severe asthma [84]. Our findings extend this evidence by further evaluating whether the choice of maintenance ICS/LABA comparator influences the estimated benefit of B/F MART. When stratified by comparator regimen, B/F MART reduced the risk of SE by 38% compared with B/F maintenance + SABA as-needed and by 25% compared with F/S maintenance + SABA as-needed.

Among ICS/LABA combinations, both Formoterol and Salmeterol are the most widely prescribed LABAs in more than 30 countries [70]. But Formoterol has pharmacological properties that make it more suitable for reliever-based use. Formoterol has been shown to be a near–full β2-agonist whereas salmeterol shows the lowest intrinsic efficacy among LABAs, consistent with its partial agonism [38]. Translating to bronchodilation, at the receptor level, Formoterol is likely to exert a greater capacity to relax airway smooth muscle than salmeterol, which may be advantageous during acute bronchoconstrictive episodes. In addition, Formoterol has a rapid onset of bronchodilation (approximately 1–3 min), whereas salmeterol has a slower onset (15–20 min) [85]. As a result, the B/F inhaler can be used for both maintenance and reliever purposes, while F/S is primarily used for maintenance therapy. Another positive finding from an in vitro study is that Formoterol portrayed a greater ability to modulate neutrophil activity than salmeterol, suggesting potential additional immunomodulatory effects when used in both maintenance and reliever regimens [86]. Although B/F MART has demonstrated favorable efficacy in reducing the risk of severe exacerbations and is widely used in many countries, B/F is approved in the U.S. as a maintenance therapy but not for as-needed or MART use [37,87]. This regulatory discrepancy may partly contribute to its variable real-world implementation. In a U.S. survey assessing MART adoption, approximately 87% of patients recommended MART were still prescribed SABA, while less than 60% of clinicians had recommended MART, suggesting persistent implementation gaps [88]. Another study evaluated the implementation of asthma regimens for moderate-to-severe asthma in the Italian healthcare system and found that although ICS-Formoterol was the most frequently reported treatment, only 21.6% of ICS-Formoterol users adopted the correct MART approach [89]. These findings suggest that, despite the clinical efficacy of B/F MART, the implementation of MART in real-world practice remains suboptimal.

For symptom relief, at present, there are only two single-inhaler therapies that combine ICS with β2-agonists: ICS adjunct with either fomoterol (LABA) or albuterol (SABA) [90,91]. Specifically, reliever-based treatment is used in mild asthma at Steps 1–2, whereas MART treatment is applied for moderate-to-severe conditions in Steps 3, 4, and 5 [92]. A 2024 review found that using ICS/Formoterol as a rescue medication reduced severe exacerbations by approximately half compared with SABA [93]. Consistently, in our study, although B/F, used as a reliever, did not reach statistical significance for the time to the first severe exacerbation or the annual severe exacerbation rate, it was associated with a ~50–60% numerical reduction over 52 weeks compared with a SABA-only strategy. It is well established that persistent asthma is underpinned by three mechanisms: T2 inflammation, airway hyper-responsiveness, and reversible airway constriction. When patients are exposed to triggers, the T2 inflammatory cascade is activated, with cytokines such as IL-4, IL-5, and IL-13 driving eosinophilic airway inflammation. As a result, airway smooth muscle becomes twitchier and more hypersensitive, making the airways increasingly prone to bronchoconstriction and hyperresponsive even to small subsequent stimuli. Accordingly, administering Budesonide whenever bronchospasm occurs may contribute to improved future bronchoprotection [94]. Given that single-inhaler B/F in our study reduced total SE episodes to a greater extent than the same dose of ICS used separately with a SABA reliever, our findings are in line with the GINA preference for the anti-inflammatory reliever (AIR) approach, where the reliever contains an ICS component combined with a bronchodilator in a single inhaler [1].

Another reason supporting the inclusion of ICS into reliever therapy is that the concurrent use of β_2_-agonists, such as LABAs or SABAs, has been associated with the development of tachyphylaxis [95]. In simpler terms, the repeated use of a LABA or SABA reduces the availability of β_2_-adrenergic receptors (β_2_AR), diminishing the bronchodilatory effects. In contrast, combining β_2_-agonists with an ICS may offset this limitation through synergistic mechanisms: the ICS can increase pulmonary β_2_AR expression, while β_2_AR activation can, in turn, enhance the anti-inflammatory effects of corticosteroids [95]. However, this should not be interpreted as encouraging patients to “switch” from SABA to B/F and then rely on it frequently, because increased reliever use is consistently associated with a higher exacerbation risk and a more persistent asthma phenotype, regardless of whether the reliever contains an ICS [96]. Thus, the key to treatment success is to minimize the frequency with which patients rely on reliever medication. Within this context, Budesonide/Formoterol has received regulatory approval as AIR therapy in individuals aged ≥12 years in over 35 countries [93], whereas the FDA has not approved B/F for as-needed treatment or MART [37,97].

As maintenance therapy, our analysis included only one of the two available RCTs, suggesting that B/F may reduce the exacerbation risk to a greater extent than Budesonide-only ICS treatment. Notably, the magnitude of evidence is relatively limited compared to the previous review [98]. Mukhopadhyay et al.’s research analyzed the B/F maintenance but on a broader scale, including 24 trials, and extended to many comparators, including ICS/LABA, B/F maintenance [98]. However, the primary outcomes assessed in that review differed from those examined in our study. Mukhopadhyay et al. focused primarily on clinical outcomes such as FEV1 improvement and asthma control, whereas we prioritized severe exacerbations, which are a key determinant in selecting the appropriate long-term regimen [98].

### 4.2. Effectiveness

While B/F MART has been shown to be superior to other therapeutic regimens in randomized trials, real-world evidence suggests it is more comparable in effectiveness to ICS/LABA + SABA. Differences in patient selection and study protocols between RCTs and non-RCTs, which also led to different risk-of-bias profiles, may have contributed to this divergent trend. It has been reported that around 5% of the real-world asthma population would meet typical RCT eligibility criteria [86,99]. RCTs tend to include a very narrow patient population, as patients are standardized during a run-in phase and required to use SABA for 5–7 days before randomization [50,70]. By enriching for patients more likely to experience exacerbations, the treatment effect of B/F versus the comparator can be captured more easily. On the other hand, non-RCTs require patients to remain under observation for at least 12 months to be included in the effectiveness analysis [41,80]. Under these conditions, the analyzed populations in both the B/F and comparator groups are less prone to exacerbations and tolerate the treatment well. Ultimately, the differences in exacerbation reduction between the two interventions become harder to discern. Moreover, in Cheng et al.’s trial, the baseline exacerbation history differed significantly between the groups [80], indicating confounding by indication. Patients receiving the comparator were initially more vulnerable to exacerbations, which could blur the true impact of B/F on exacerbation outcomes and make the results appear more comparable.

Another difference in B/F efficacy between RCTs and non-RCTs is that findings from randomized trials compare the efficacy of treatment strategies, rather than isolating the intrinsic effectiveness of individual therapeutic components. This contrast is less apparent in non-RCTs, where B/F was compared with other ICS/LABA options within clinical use (maintenance vs. maintenance, MART vs. MART). Recent studies have observed greater efficacy in reducing exacerbation rates with FF/VIL than with B/F as a maintenance strategy [100,101,102]. Our findings extend this evidence by suggesting that this apparent advantage does not necessarily translate to the MART setting, where the two interventions showed comparable efficacy. This observation is notable given that, to date, only two regimens are authorized for MART usage: Budesonide/Formoterol and Beclomethasone/Formoterol [92,103]. Nevertheless, the interpretation should be taken with caution as only one trial was synthesized. Within the ICS class, fluticasone furoate has been proven to have the highest anti-inflammatory effect [94,104,105]. Its effect is up to 24h, allowing sustained bronchoprotection and once-daily dosing, in contrast to the twice-daily regimen typically required for Budesonide. Furthermore, Vilanterol is among the ultra-long-acting LABAs; thus, its combination with fluticasone furoate is expected to provide greater reductions in exacerbations and reliever use, potentially supporting better treatment adherence compared to B/F [100,106,107].

A simulation study comparing B/F twice daily and FF/VIL once daily in moderate-to-severe asthma found that B/F MART would require an additional 3 to 7.25 inhalations compared with FF/VIL [101]. Lower levels of cortisol suppression were also observed in the FF/VIL group, indicating potentially reduced systemic corticosteroid exposure [101]. The dosing simplicity, as well as the prolonged bronchodilatory profile of Fluticasone furoate/Vilanterol, may help FF/VIL numerically improve overall asthma control compared to B/F (higher ACT scores, but not reaching the minimal clinically important difference) [41]. Furthermore, MART regimens have been associated with a high rate of uncontrolled asthma symptoms, with approximately 44.3% of patients remaining uncontrolled. Despite reported adherence, patients receiving MART may still experience severe exacerbations within the first year after treatment initiation [108]. In a 12-week post-marketing study in India evaluating FF/VI as a maintenance therapy in patients uncontrolled on conventional treatment, most patients had previously been treated with ICS/LABA, with B/F SMART being the most common baseline regimen. Treatment with FF/VI led to significant improvement in asthma symptoms, as reflected by a reduction in the ACQ-5 score. Notably, no severe exacerbations were recorded during the 12-week treatment period [108]. Thus, although FF/VIL is not approved for MART use, it may serve as a complementary once-daily maintenance option for selected patients who remain uncontrolled on MART.

The increasing role of biologics is defined by their ability to provide targeted interventions based on predictive biomarkers and specific clinical phenotypes [44]. Anti-IgE therapy (omalizumab) was the first biologic approved for asthma, specifically targeting perennial allergen sensitization and reducing seasonal viral exacerbations [43,44]. For patients with severe eosinophilic asthma, anti-interleukin-5 (anti-IL-5) agents like mepolizumab and reslizumab, or the receptor blocker benralizumab, effectively reduce severe exacerbations by depleting blood and tissue eosinophils [43,44]. Anti-IL-4/13 (dupilumab) blocks the IL-4Rα subunit, demonstrating high efficacy in reducing oral corticosteroid (OCS) dependence and managing Type 2 comorbidities such as nasal polyps and atopic dermatitis [43,44]. A major advancement is the introduction of anti-TSLP (tezepelumab), which targets epithelial alarmins and maintains clinical efficacy irrespective of T2 biomarker status, providing a vital option for T2-low patients [44]. Selecting the optimal biologic is a highly individualized process that requires clinicians to integrate blood eosinophil counts, fractional exhaled nitric oxide (FeNO), and total IgE with a patient’s unique comorbidities and OCS requirements [43,44]. Through these personalized mechanisms, modern therapy now strives toward achieving clinical remission and minimizing the long-term adverse effects associated with high-dose steroid use [43,44].

### 4.3. Safety

Our findings indicated that Budesonide/Formoterol is well tolerated as a maintenance, reliever, or combined therapy (MART), with comparable incidences of AEs, SAEs, and discontinuation rates across comparisons. This was observed at both the low dose (200–400 µg/day) and the medium dose (>400–800 µg/day) of B/F, with dosage levels determined based on the total daily dose of ICS (e.g., Budesonide) for adolescents and adults [109]. The most common AEs in the B/F groups were upper respiratory tract issues (e.g., infections, nasopharyngitis, and influenza), with no significant differences compared with the other groups. These symptoms are known to be associated with ICS use [110,111] and align with the findings from previous studies on the safety of B/F [48,98].

Regarding the safety of B/F MART, although the overall AEs rates were substantial, B/F MART demonstrated lower AE-related discontinuation rates than conventional ICS-containing maintenance plus as-needed SABA regimens over 24–52 weeks [39,72,76,78]. The observed gap of around 20% in AE rates between B/F MART (38–61.4%) and B/F reliever (38–88%) over 52 weeks is expected, given the mandatory daily maintenance dose in the MART regimen. However, this regular ICS exposure with B/F MART does not necessarily translate into higher cumulative corticosteroid toxicity. Following our included RCTs, compared to ICS-containing therapies (ICS maintenance; ICS/LABA + SABA), the mean total daily ICS dose was 25–62.5% lower in the B/F MART group over a 6- to 12-month treatment period [35,50,72,73]. This observation appears to be closely linked to the reduction in severe exacerbations (SE). A lower frequency of exacerbation events with B/F MART may reduce the subsequent need for rescue medications, thereby lowering cumulative corticosteroid exposure. This trend is compatible with previous studies, which suggest an association between B/F MART and a decreased requirement for oral corticosteroids [42,45].

Adherence still significantly affects treatment efficacy in adolescent and adult patients with asthma, especially since concerns about adverse events are a primary barrier to adherence [112,113]. Real-world data indicate that non-adherence to ICS was significantly higher in lower GINA steps, peaking at 82% in Step 2, with approximately one-third of patients with mild asthma (Steps 1–2) reporting poor adherence [114,115]. In the current review, our analysis revealed that the AEs and AE-related discontinuation rates were similar between as-needed B/F and alternative regimens (e.g., as-needed SABA or ICS maintenance plus as-needed SABA using two separate inhalers) in patients with mild asthma [51,70,71], although slightly lower in the B/F group [51,70,71]. Based on these findings and the comparable efficacy across regimens, using as-needed B/F from the outset (Step 1) simplifies device use, thereby improving adherence and reducing the need for OCS by lowering the risk of exacerbation. These observations further support the current treatment regimen in adolescents and adults, specifically the recommendation for as-needed B/F in Steps 1–2 of Track 1 [109].

Additionally, our results did not show any abnormal AEs or mortality due to B/F use, thereby supporting previous studies on the consistent safety of B/F compared to SABA alone or the ICS plus as-needed SABA regimen [46,116]. However, there was substantial evidence indicating that the overuse of SABA is a significant risk factor for exacerbations and asthma-related mortality [24,25]. To mitigate this risk, B/F offers a distinct pharmacological advantage over the SABA reliever regimen. While SABA only provides bronchodilation [117], which potentially leaves the underlying inflammation uncontrolled, as-needed B/F ensures that airway inflammation is addressed simultaneously with every symptom-relief actuation. Previous studies also highlighted that the MART strategy effectively reduces SABA reliance and helps prevent the progression of severe asthma exacerbations [42,118]. Overall, these observations further validate the recommendation to use B/F MART in Steps 3–4 [109] This strategy effectively eliminates the overuse of SABA, ensuring that any increase in symptoms is met with the necessary anti-inflammatory intervention.

Furthermore, in line with previous studies [77,119], our analysis found an extremely low incidence (ranging 0.5–2.0%) of both Formoterol-related cardiovascular adverse events (e.g., tachycardia, palpitations, arrhythmogenic potential) and ICS-associated complications (e.g., oropharyngeal candidiasis, dysphonia). However, a comprehensive review by Rayner et al. (2024) [120] on ICS/Formoterol therapy still reported a non-significant incidence of severe CDV events and pneumonia over a 6- to 54-week period, whereas our analysis did not identify any treatment-related events of this nature [51,70]. This discrepancy is likely attributable to methodological constraints, as the RCTs included in our study generally had modest sample sizes and were not adequately powered to detect these rare harms within a limited 52-week timeframe. Therefore, these cumulative risks still require careful monitoring in routine practice.

Clinically relevant cardiovascular adverse effects associated with long-acting β2-agonist (LABA)-containing therapies are recognized as pharmacologically predictable class effects of β2-adrenoceptor stimulation [72,74,75,78]. These adverse events primarily manifest as tachycardia, palpitations, and arrhythmias, including specific reports of atrial fibrillation [72,74,78]. Systematic reviews and randomized controlled trials consistently demonstrate an extremely low incidence of these events, typically ranging from 0.5% to 2.0% in patients treated with combinations such as Budesonide/Formoterol [72,73,74,78]. Despite their rarity, cardiovascular symptoms like palpitations are among the most frequently cited class-related reasons for treatment discontinuation in clinical studies [50,73,76]. There is a theoretical concern that the use of highly efficacious agonists could lead to greater cardiotoxicity—specifically tachyarrhythmias—during episodes of extreme overuse, particularly when exacerbated by the hypoxia present during a severe asthma attack [38,42,76]. However, comprehensive pooled analyses and network meta-analyses of large-scale clinical trials have confirmed that LABAs, when used in fixed-dose combinations with inhaled corticosteroids, do not significantly increase the risk of serious cardiovascular adverse events or mortality compared with short-acting β2-agonists or inhaled corticosteroids alone [36,98,120].

Inhaled corticosteroids (ICS) are fundamental to asthma management and are generally well-tolerated, though they are associated with several specific local and systemic adverse effects [110]. The most common side effects are respiratory-related, specifically upper respiratory tract infections (URTIs), nasopharyngitis, pharyngitis, and bronchitis [35,40,48,51,110]. Local complications resulting from drug deposition in the throat include oropharyngeal candidiasis (oral thrush) and dysphonia (hoarseness or voice changes), which typically occur at a low incidence of 0.5% to 2.0% but can increase with higher doses [72,73,74,78]. Other frequently reported symptoms include a sore throat, a cough, pharyngolaryngeal pain, and dysgeusia (adverse taste) [51,72,98,112]. Although the systemic effects are more prevalent with oral corticosteroids, long-term or high-dose ICS exposure can lead to cortisol suppression, skin thinning, bruising, and potentially increased risks of cataracts, glaucoma, and diabetes (hyperglycemia) [101,104,110,112]. Furthermore, at very high cumulative doses, there are concerns regarding bone mass attrition and an increased risk of fractures [98,101]. To minimize these risks, clinicians recommend preventive measures such as using spacer devices or rinsing the mouth with water after inhalation to reduce local drug deposition [98,112].

### 4.4. Implications, Strengths, and Limitations

The findings from our study suggest relevant clinical implications, particularly for patients with moderate-to-severe asthma. Regarding asthma control, B/F therapy is superior to ICS/LABA + SABA regimens in RCTs, although efficacy is often comparable in real-world settings (non-RCTs). Specifically, in RCTs, our analysis highlights the superior efficacy of the B/F MART regimen compared with maintenance therapies (either B/F or F/S) combined with as-needed SABA. Concurrently, safety data reinforce that B/F has a well-tolerated profile, demonstrating non-inferiority to current ICS/LABA + SABA regimens. Drawing upon these three key observations, B/F might be an effective and safe alternative over a 12-month period for patients with moderate-to-severe asthma, particularly those with poor treatment adherence or at an elevated risk of severe exacerbations. For patients with more severe symptoms associated with T2 inflammation who remain uncontrolled despite dual therapy, more potent approaches such as biologic-based precision medicine may be appropriate [121,122,123]. However, treatment responses remain variable, and many patients remain uncontrolled. T2-low disease lacks good options, and cost/access and biomarker limitations prevent it from being a universal or standalone solution for broad asthma management [43,44].

This systematic review’s main strength is its comprehensive focus on long-term clinical trials (e.g., 6–12 months), evaluating both B/F MART and B/F as maintenance or reliever monotherapy. Including studies with up to 12 months of follow-up, such as recent RCTs and real-world data, provides a thorough evaluation of the severe exacerbation risk across diverse treatment strategies. To the best of our knowledge, this is one of the few comprehensive studies to evaluate the AE-related factors of B/F across all three regimens. This helps clarify earlier assessments of the safety and potential to reduce the risk of adverse events associated with B/F compared with other ICS/LABA or SABA-only regimens [46,116,120]. While the systematic review indicates that Budesonide/Formoterol (B/F) is “well-tolerated,” this conclusion is limited by the 6- to 12-month duration of the included clinical trials. Because asthma is a lifelong chronic condition, these randomized controlled trials (RCTs) have inherent limitations and may not accurately reflect the chronic safety profile or very long-term tolerability of a treatment that patients often utilize for many years [116]. Most available outcome data do not extend beyond 52 weeks, so the cumulative effects of decades of maintenance therapy remain under-researched for these specific regimens analyzed in this meta-analysis [46,116]. Consequently, the potential for long-term adverse events associated with chronic ICS or Formoterol exposure—such as cumulative systemic effects or impacts on lung function decline—still requires vigilant monitoring in real-world clinical practice. Short-term study designs are often insufficient to evaluate complex, slowly progressing outcomes like airway remodeling or sustained changes in lung function [36,116]. To bridge this gap, future research must prioritize large-scale longitudinal studies and real-world registry data to fully assess the chronic tolerability and sustainability of B/F-based strategies over a patient’s entire lifespan [36,46,116].

However, our review also has certain limitations. Most trials focus on adolescent and adult populations. Although numerous studies have evaluated the efficacy of B/F in asthma management, the real-world effectiveness of B/F in children under 11 years of age remains limited. This gap can complicate the determination of the optimal initial treatment strategy for this younger demographic. Compared to the MART strategy, the evidence supporting the use of B/F as needed or as maintenance monotherapy in this review is considerably less robust. While 14 RCTs support the efficacy of MART, only four studies have examined B/F as-needed monotherapy, and only two reports have compared maintenance B/F with Budesonide alone. This limited amount of data may not be sufficient to draw clear or comprehensive conclusions about the long-term effectiveness and safety of these monotherapy regimens compared with other treatment options. Therefore, more extensive future studies are necessary to thoroughly assess B/F’s treatment profile outside the MART context.

Nonetheless, our conclusions are based solely on RCTs, which have inherent limitations in representing a lifelong chronic condition such as asthma. Because the majority of the included trials had a limited follow-up of 6- to 12-month periods, our ability to definitively assess the chronic safety profile and very long-term tolerability of B/F remains limited, as these short-term studies may not accurately reflect real-world safety. Therefore, long-term observational studies and real-world registry data remain essential for monitoring the chronic tolerability of B/F over time.

## 5. Conclusions

Budesonide/Formoterol as a MART regimen is the most effective for preventing severe exacerbations compared with ICS/LABA + SABA, ICS + SABA, and is not inferior to conventional best practice. In longer-duration studies, as-needed B/F remained favorable over a SABA-only reliever therapy in reducing the severe exacerbation risk, although this advantage was not statistically significant when compared with ICS maintenance + as-needed SABA. Evidence comparing B/F as a maintenance dose vs. an ICS-only strategy remained limited. Real-world studies, although limited, suggest that B/F demonstrates effectiveness in reducing exacerbation rates comparable to that of comparators. Over 6- to 12-month treatment, Budesonide/Formoterol is well tolerated. Nevertheless, considering the lifelong trajectory of asthma, the treatment and AEs associated with long-term ICS or Formoterol still require regular monitoring in a real-world clinical context.

## 6. Future Directions

Future research on Budesonide/Formoterol (B/F) in asthma should prioritize narrowing the gap between randomized controlled trial (RCT) efficacy and real-world effectiveness through pragmatic trials and large-scale longitudinal studies, particularly given the current evidence supporting the superiority of B/F as maintenance and reliever therapy (MART) in reducing severe exacerbations while still demonstrating heterogeneity across patient populations. A key direction involves advancing phenotype- and biomarker-driven approaches to better stratify patients by inflammatory endotypes (e.g., type 2-high vs. type 2-low asthma), thereby optimizing the selection among MART, fixed maintenance, or reliever-based strategies. In parallel, further pharmacokinetic–pharmacodynamic (PK/PD) investigations and real-world adherence analyses are essential to refine dosing flexibility and inhaler use, particularly in populations with suboptimal inhalation technique or adherence, which remain critical determinants of clinical outcomes. Beyond efficacy, future work should integrate health economic evaluations to assess the cost-effectiveness relative to emerging therapies such as biologics and triple therapies, especially in the context of exacerbation-driven healthcare expenditures. The incorporation of digital health technologies, including smart inhalers and real-time monitoring systems, may further enhance treatment effectiveness by optimizing adherence and enabling the early detection of exacerbations. Finally, long-term safety surveillance and comparative effectiveness studies across diverse and underrepresented populations are necessary to ensure the generalizability and sustainability of B/F-based strategies within evolving global asthma management frameworks.

## Figures and Tables

**Table 1:** Characteristics of qualified studies.

No	Author, Year, Ref.	Study Design	Trial ID	Age	Asthma Severity, %FEV1 Requirement and Mean Baseline	B/F Indication	Comparison	Metered Dose	Efficacy Sample	Type of Outcome	Conduction Place	Funding
1	O’Byrne et al., 2018 [70]	RCT	NCT02149199	≥12	Mild, mean FEV_1_ = 84%	Reliever	PLB + B/F vs. PLB + TEB vs. BUD + TEB	PLB + 200/6 μg vs. PLB + 0.5 mg vs. 200 μg + 0.5 mg	3836	Efficacy, Safety	18 countries	AstraZeneca
2	Beasley et al., 2019 [51]	RCT	ACTRN12615000999538	18–75	Mild, mean FEV_1_ = 89%	Reliever	B/F vs. ALB vs. BUD + ALB	200/6 μg vs. 100 μg vs. 200 μg + 100 μg	668	Efficacy, Safety	New Zealand, the United Kingdom, Italy, and Australia	AstraZeneca and the Health Research Council of New Zealand
3	Bateman et al., 2018 [71]	RCT	NCT02224157	≥12	Mild, mean FEV_1_ = 84.3%	Reliever	PLB + B/F vs. BUD + TEB	PLB + 200/6 μg vs. 200 μg + 0.5 mg	4176	Efficacy, Safety	25 countries	AstraZeneca
4	Hardy et al., 2019 [79]	RCT	ACTRN12616000377437	18–75	Mild-to-moderate, mean FEV_1_ = 87–88%	Reliever	B/F vs. BUD + TEB	200/6 μg vs. 200 μg + 250 μg	885	Efficacy, Safety	New Zealand	Health Research Council of New Zealand
5	Lalloo et al., 2003 [75]	RCT	-	≥18	Mild-to-moderate, FEV_1_ 60–90%, mean FEV1= 82%	Maintenance	B/F vs. BUD	80/4.5 μg vs. 200 μg	467	Efficacy, Safety	7 countries *	AstraZeneca, GSK, Novartis, and Merck
6	Peters et al., 2016 [77]	RCT	NCT01444430	≥12	Moderate-to-severe	Maintenance	B/F vs. BUD	80/4.5 μg vs. 80 μg 160/4.5 μg vs. 160 μg	11,693	Efficacy, Safety	25 countries	AstraZeneca
7	Bousquet et al., 2007 [50]	RCT	D589 0C00002	≥12	Moderate-to-severe persistent, FEV_1_ ≥ 50%, mean FEV_1_ = 70%	MART	B/F vs. F/S + TEB	160/4.5 μg vs. 50/500 μg + 0.4 mg	2304	Efficacy, Safety	17 countries	AstraZeneca
8	Vogelmeier et al., 2005 [39]	RCT	-	≥12	Moderate persistent, FEV_1_ 40–90%, mean FEV_1_ = 73%	MART	B/F vs. F/S + SAB	160/4.5 μg vs. 250/50 μg + 100 μg	2135	Efficacy, Safety	16 countries	AstraZeneca
9	Kuna et al., 2007 [35]	RCT	SD-039-0735	≥12	Moderate-to-severe FEV_1_ ≥ 50%, mean FEV_1_ = 72–73%	MART	B/F vs. F/S + TEB vs. B/F + TEB	160/4.5 μg vs. 125/25 μg + 0.4 mg vs. 320/9 μg + 0.4 mg	3335	Efficacy, Safety	16 countries	AstraZeneca
10	O’Byrne et al., 2005 [73]	RCT	-	4–80	Moderate-to-severe, FEV_1_ 60–100%, mean FEV_1_ = 73%	MART	B/F vs. B/F + TEB vs. BUD + TEB	80/4.5 μg vs. 80/4.5 μg + 0.4 mg vs. 320 μg + 0.4 mg	2760	Efficacy, Safety	22 countries	AstraZeneca
11	Atienza et al., 2013 [40]	RCT	NCT00839800	≥16	Moderate-to-severe, FEV_1_ ≥ 50%, mean FEV_1_ = 70%	MART	B/F vs. B/F + TEB	160/4.5 μg vs. 160/4.5 μg + 0.4 mg	2091	Efficacy, Safety	13 countries	AstraZeneca
12	Patel et al., 2013 [76]	RCT	ACTRN12610000515099	16–65	Moderate-to-severe asthma, mean FEV_1_ = 82%	MART	B/F vs. B/F+ SAB	200/6 μg BID vs. 200/6 μg BID + 100 μg	303	Efficacy, Safety	New Zealand	Health Research Council of New Zealand
13	Scicchitano et al., 2004 [72]	RCT	-	12–80	Moderate-to-severe, FEV_1_ 50–90%, mean FEV_1_ = 70%	MART	B/F vs. BUD + TEB	160/4.5 μg vs. + 160 μg + 0.4 mg	1890	Efficacy, Safety	18 countries	AstraZeneca
14	Rabe et al., 2006 [74]	RCT	-	12–80	Mild-to-moderate, FEV_1_ 60–100%, mean FEV_1_ = 75%	MART	B/F vs. BUD + TEB	80/4.5 μg vs. 160 μg + 0.4 mg	695	Efficacy, Safety	16 countries	AstraZeneca
15	Rabe et al., 2006 [78]	RCT	-	≥12	Moderate-to-severe, FEV_1_ 50–100%, mean FEV_1_ = 72%	MART	B/F vs. B/F + FOR vs. B/F + TEB	160/4.5 μg vs. 160/4.5 μg +4.5 μg vs. 160/4.5 μg +0.4 mg	3392	Efficacy, Safety	20 countries	AstraZeneca
16	Huang et al., 2024 [41]	Non-RCT	-	≥20	Moderate-to-severe (step 3,4 GINA), mean FEV_1_ = 88.8–91.6%	MART	B/F vs. FF/VIL MART	B/F vs. 92/22 μg or 184/22 μg	161	Effectiveness	Taiwan	-
17	Cheng et al., 2020 [80]	Non-RCT	NCT00784953	-	Moderate-to-severe	MART	B/F vs. F/S + SABA	-	723	Effectiveness	Taiwan	AstraZeneca
18	Hanania et al., 2025 [81]	Non-RCT	-	≥18	Moderate-to-severe, mean FEV_1_ = 85–86%	Maintenance	B/F vs. F/S	80/4.5 μg, 160/4.5 μg vs. 100/50 μg, 250/50 μg	57,000	Effectiveness	USA	GSK
19	Tunceli et al., 2014 [82]	Non-RCT	NCT01623544	12–64	Moderate-to-severe	Maintenance	B/F vs. F/S	80/4.5 μg, 160/4.5 μg BID vs. BID^dose^	6086	Effectivenes	USA	AstraZeneca

Abbreviation: ALB: Albuterol; BUD: Budesonide; B/F: Budesonide/Formoterol; F/S: Fluticasone propionate/Salmeterol; FF/VIL: Fluticasone furoate/Vilanterol; FEV1: Forced Expiratory Volume in 1 s; GINA: Global Initiative for Asthma; MART: Maintenance and Reliever Therapy; Non-RCT: Non-randomized Controlled Trial; RCT: Randomized Controlled Trial; PLB: Placebo; SAB: Salbutamol; SABA: Short-Acting β2-Agonist; TEB: Terbutaline; *: The Czech Republic, Hungary, Norway, Poland, South Africa, Sweden, and the United Kingdom; ^dose^: 100/50 μg, 250/50 μg, 500/50 μg for powder, 45/21 μg, 115/21 μg, 230/21 μg for aerosol.

**Table 2:** Severe exacerbation events and FEV1, ACQ-5/ACT assessment between Budesonide/Formoterol as MART or maintenance therapy in a non-RCT.

Author, Year	Duration	Comparison	Annual Exacerbation Rate ***	Hospitalization Rate Due to Exacerbation ***	FEV1 in Liter		Asthma Control Score (ACQ-5/ACT)		Conclusion
					Mean Baseline (SD)	Mean Change (SD)	Mean Baseline	Mean Change	
**MART: B/F vs. ICS/LABA + SABA**									
Cheng et al., 2020 [80]	16 weeks	B/F vs. F/S + SABA as-needed	0.373 vs. 0.349 *, *p* > 0.05	0.036 vs. 0.027, *p* > 0.05	1.82 ± 0.77 vs. 1.54 ± 0.72	0.09 ± 0.36 vs. −0.01 ± 0.48, *p* = 0.420	1.54 ± 1.06 vs. 1.46 ± 1.28	−0.91 ± 1.11 vs. −0.69 ± 1.27, *p* = 0.027	B/F improves lung function and asthma control, not inferior to F/S+SABA
**MART: B/F vs. ICS/LABA+ ICS/LABA**									
Huang et al., 2024 [41]	52 weeks	B/F BID vs. FF/VIL 92/22 μg, 184/22 μg OD + FF/VIL as-needed	−0.14 vs. −0.04, *p* = 0.492 **	-	88.8 (81.9, 95.7) vs. 91.6 (88.5, 94.9) ^p^	−0.06 (−4.35, 4.23) vs. 1.26 (–1.58, 4.11), *p* = 0.186	23 vs. 22	0.88 vs. 1.57, *p* < 0.001	B/F is as effective as F/V in MART
**Maintenance: B/F vs. ICS/LABA**									
Hanania et al., 2025 [81]	12 months	B/F 80/4.5 μg, 160/4.5 μg BID vs. F/S 100/50 μg, 250/50 μg BID	0.40 vs. 0.38, *p* = 0.092	-	-	-	-	-	B/F is effective as F/S in reducing exacerbation rate
Tunceli et al., 2014 [82]	12 months	B/F 80/4.5 μg, 160/4.5 μg BID vs. F/S BID dose	0.85 vs. 0.93, *p* = 0.0255	0.01 vs. 0.01, *p* = 0.6390	-	-	-	-	B/F is more effective than F/S in reducing exacerbation rate

Abbreviation: ACQ-5: Asthma Control Questionnaire; ACT: Asthma Control Test; MART: Maintenance and Reliever Therapy; B/F: Budesonide/Formoterol; F/S: Fluticasone propionate/Salmeterol; FF/VIL: Fluticasone furoate/Vilanterol; BID: Twice-Daily; OD: Once-daily; inh: inhalation; ICS: Inhaled Corticosteroids; LABA: Long-Acting β2-Agonist; SE: Severe exacerbation; SABA: Short-Acting β2-Agonist; *: derived value; **: mean change in annual exacerbation; ***: value displayed as event/patient/year.

**Table 3:** Adverse event associated with Budesonide/Formoterol as MART.

Authors, Year, N	Duration	Maintenance Dose	AEs				SAEs (%)
			Incidence (%)	Severity	Most Common Symptoms	Discontinuation Rate (%)	
**vs. ICS/LABA maintenance + SABA as-needed**							
O’Byrne et al., 2005, 922 vs. 906 [73]	52 weeks	B/F 80/4.5 μg BID vs. B/F 80/4.5 μg BID + TEB as-needed	54 vs. 52	Mild-to-moderate	-	-	5 vs. 7
Rabe et al., 2006, 1107 vs. 1137 vs. 1138 [78]	52 weeks	B/F 160/4.5 μg × 1 inh BID vs. B/F 160/4.5 μg + FOR as-needed vs. B/F 160/4.5 μg + TEB as-needed	-	-	Candidosis, hoarseness, palpitation, tremor	1.1 vs. 1.9 vs. 1.7	1 vs. 2 vs. 2 *
Atienza et al., 2013, 1049 vs. 1042 [40]	52 weeks	B/F 160/4.5 μg × 1 inh BID vs. B/F 160/4.5 μg × 1 inh BID + TEB as-needed	57.4 vs. 57.5	Mild-to-moderate	Nasopharyngitis, bronchitis, and viral upper respiratory tract infection in both groups	-	4.0 vs. 7.1
Kuna et al., 2007, 1105 vs. 1123 [35]	26 weeks	B/F 160/4.5 μg *×* 1 inh BID vs. B/F 320/9 μg *×* 1 inh BID + TEB as-needed	-	-	Upper respiratory tract infection, pharyngitis, and nasopharyngitis in all groups	-	3 vs. 4
		B/F 160/4.5 μg × 1 inh BID vs. F/S 125/25 μg × 2 inh BID + TEB as-needed	-	-		-	3 vs. 3
Vogelmeier et al., 2005, 1067 vs. 1076 [39]	52 weeks	B/F 160/4.5 μg *×* 2 inh BID vs. F/S 250/50 μg BID + SAB as-needed	-	-	-	2.53 vs. 2.60 **	7.5 vs. 8.18 **
Bousquet et al., 2007, 1144 vs. 1145 [50]	26 weeks	B/F 160/4.5 μg × 2 inh BID vs. F/S 250/50 μg BID + TEB as-needed	39 vs. 40	-	-	-	3 vs. 3
Patel et al., 2013, 151 vs. 152 [76]	24 weeks	B/F 160/4.5 μg × 2 inh BID vs. B/F 160/4.5 μg × 2 inh BID + SAB as-needed	Similar between groups	-	Upper respiratory tract infection, adverse taste	<1 vs. 1	5.3 vs. 4.6
**B/F vs. ICS maintenance + SABA as-needed**							
Rabe et al., 2006, 354 vs. 342 [74]	26 weeks	BF 80/4.5 μg *×* 2 inh OD vs. BUD 160 μg *×* 2 inh OD + TEB as-needed	38 vs. 41	Mild-to-moderate	Respiratory infection in both groups	-	-
O’Byrne et al., 2005, 922 vs. 925 [73]	52 weeks	BF 80/4.5 μg BID vs. BUD 320 μg BID + TEB as-needed	54 vs. 57	Mild-to-moderate	-	-	5 vs. 5
Scicchitano et al., 2004, 947 vs. 943 [72]	52 weeks	BF 160/4.5 μg *×* 2 inhs OD vs. BUD 160 μg *×* 2 inh BID + TEB as-needed	56 vs. 57	Mild-to-moderate	Respiratory infection, bronchitis, Aggravated asthma in both groups	3 vs. 4	-

Abbreviation: MART: Maintenance and Reliever Therapy; B/F: Budesonide/Formoterol; F/S: Fluticasone propionate/Salmeterol; BUD: Budesonide; TEB: Terbutaline; FOR: Formoterol; SAB: Salbutamol; OD: Once-Daily; BID: Twice-Daily; AE: Adverse Event; SAE: Serious Adverse Event; ICS: Inhaled Corticosteroids; inh: inhalation; SABA: Short-Acting β2-Agonist; LABA: Long-Acting β2-Agonist. * SAE related to asthma; **: derived value.

**Table 4:** Adverse event between Budesonide/Formoterol as Reliever or Maintenance therapy.

Authors, Year	Duration	Comparison	N	AEs			SAEs (%)
				Incidence (%)	Most Common	Discontinue Rate (%)	
**Reliever:** **B/F vs. SABA**							
O’Byrne et al., 2018 [70]	52 weeks	PLB BID + B/F 200/6 μg as-needed vs. PLB BID + TEB 0.5 mg as-needed	1277 vs. 1277	38.0 vs. 42.7	Upper respiratory tract infection, viral upper respiratory tract infection, and asthma in all groups	0.8 vs. 2.9	3.0 vs. 3.9
Beasley et al., 2019 [51]	52 weeks	B/F 200/6 μg as-needed vs. ALB 100 μg × 2 inh as-needed	220 vs. 223	78.4 vs. 81.9	Upper respiratory infection, nasopharyngitis, influenza vs. Upper respiratory tract infection, nasopharyngitis, asthma	-	5.5 vs. 2.7
**Reliever:** **B/F vs. ICS maintenance + SABA**							
Beasley et al., 2019, 220 vs. 225 [51]	52 weeks	B/F 200/6 μg as-needed vs. BUD 200 μg BID + ALB 100 μg × 2 inh as-needed	220 vs. 225	78.4 vs. 83.7	Upper respiratory infection, nasopharyngitis, influenza vs. Upper respiratory tract infection, nasopharyngitis, asthma	-	5.5 vs. 2.6
O’Byrne et al., 2018 [70]	52 weeks	PLB BID + B/F 200/6 μg as-needed vs. BUD 200 μg BID + TEB 0.5 mg as-needed	1277 vs. 1282	38.0 vs. 39.9	Upper respiratory tract infection, viral upper respiratory tract infection, and asthma in all groups	0.8 vs. 1.2	3.0 vs. 2.9
Bateman et al., 2018 [71]	52 weeks	PLB BID + B/F 160/4.5 μg as-needed vs. BUD 200 μg BID + TEB 0.5 mg as-needed	2089 vs. 2087	42.5 vs. 44.0	Viral upper respiratory tract infection, asthma, upper respiratory tract infection in both groups	0.7 vs. 1.1	3.2 vs. 3.5
Hardy et al., 2019 [79]	52 weeks	B/F 160/4.5 μg as-needed vs. BUD 200 μg BID + TEB 0.5 mg as-needed	437 vs. 448	88 vs. 83	Nasopharyngitis in both groups	-	-
**Maintenance: B/F vs. ICS**							
Lalloo et al., 2003 [75]	12 weeks	B/F 80/4.5 μg BID vs. BUD 200 μg BID	-	-	Respiratory infection, pharyngitis, and rhinitis in both groups	-	-
Peters et al., 2016 [77]	26 weeks	B/F 80/4.5–160/4.5 μg vs. BUD 80–160 μg	5486 vs. 5487	-	-	1.6 vs. 2.3	2.1 vs. 2.1

Abbreviation: AE: Adverse Event, ALB: Albuterol; B/F: Budesonide/Formoterol; F/S: Fluticasone propionate/Salmeterol; BUD: Budesonide; TEB: Terbutaline; FOR: Formoterol; BID: Twice-Daily; PLB: Placebo; SAE: Serious Adverse Event; ICS: Inhaled Corticosteroids; inh: inhalation; SABA: Short-Acting β2-Agonist; LABA: Long-Acting β2-Agonist.

## Data Availability

The data were collected from PubMed, Embase, and Cochrane.

## Supplementary Materials

The following supporting information can be downloaded at: https://www.mdpi.com/article/10.3390/healthcare14131864/s1.
