# Supplementary material for: Effectiveness and Safety of Budesonide/Formoterol in Asthma: A Systematic Review
Source: Healthcare (Basel). 2026 Jun 26;14(13):1864. doi: 10.3390/healthcare14131864 (PMC13360979; doi:10.3390/healthcare14131864)
Supplement: Supplementary file 1 [file healthcare-14-01864-s001.zip › Supplemtal.pdf]

Systematic Review

Efficacy, Effectiveness, and Safety of Budesonide/Formoterol in Asthma: A Systematic Review

2. Materials and Methods

2.1. Searching strategy

Table S1. Multiple attempts were made to utilize the syntax to retrieve research identification from the PubMed, Cochrane, and Embase databases.

No.	Syntax	Number of results
	**Pubmed**	
1	(Asthma) AND ((Budesonide) AND (Formoterol) OR (Formoterol fumarate))) AND ((Efficacy) OR (Effectiveness) OR (Safety))	725
2	((budesonide) AND ((formoterol) OR (formoterol fumarate))) AND (asthma) AND (exacerbation*) AND ((efficacy) OR (effectiveness) OR (safety))	303
3	((budesonide) AND ((formoterol) OR (formoterol fumarate))) AND ((vilanterol) OR (fluticasone) OR (albuterol) OR (salmeterol) OR (salbutamol)) AND (asthma) AND ((efficacy) OR (effectiveness) OR (safety))	299
4	(budesonide) AND ((formoterol) OR (formoterol fumarate)) AND (asthma) AND ((severe exacerbation) OR (first time severe exacerbation) OR (SE)) AND ((efficacy) OR (effectiveness) OR (safety))	226
	**Cochrane**	
1	(Asthma) AND ((Budesonide) AND ((Formoterol) OR (Formoterol fumarate))) AND ((Efficacy) OR (Effectiveness) OR (Safety))	585
2	(budesonide) AND ((formoterol) OR (formoterol fumarate)) AND (asthma) AND ((severe exacerbation) OR (first time severe exacerbation) OR (SE)) AND ((efficacy) OR (effectiveness) OR (safety))	190
	**Embase**	
1	(Asthma) AND ((Budesonide) AND (Formoterol) OR (Formoterol fumarate)) AND ((Efficacy) OR (Effectiveness) OR (Safety))	2454
2	(budesonide) AND ((formoterol) OR (formoterol fumarate)) AND (asthma) AND ((severe exacerbation) OR (first time severe exacerbation) OR (SE)) AND ((efficacy) OR (effectiveness) OR (safety))	386

3. Results

3.3. Risk of bias assessment

3.3.1. RoB 2.0 tool for evaluating RCT

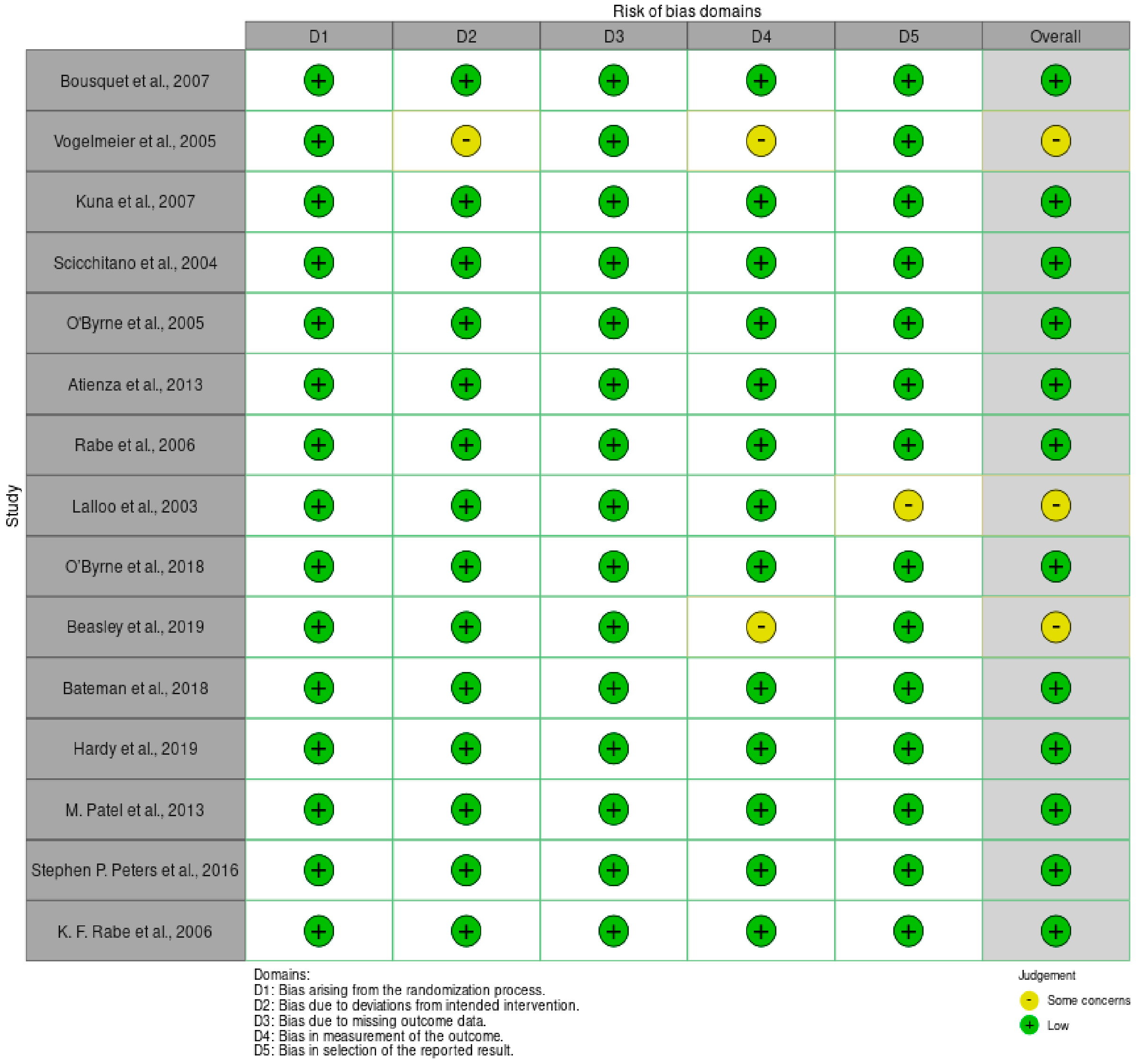

Figure S1. Risk of bias assessment for the included randomized controlled trials.

3.3.2. ROBINS-I tool for evaluating Non-RCT

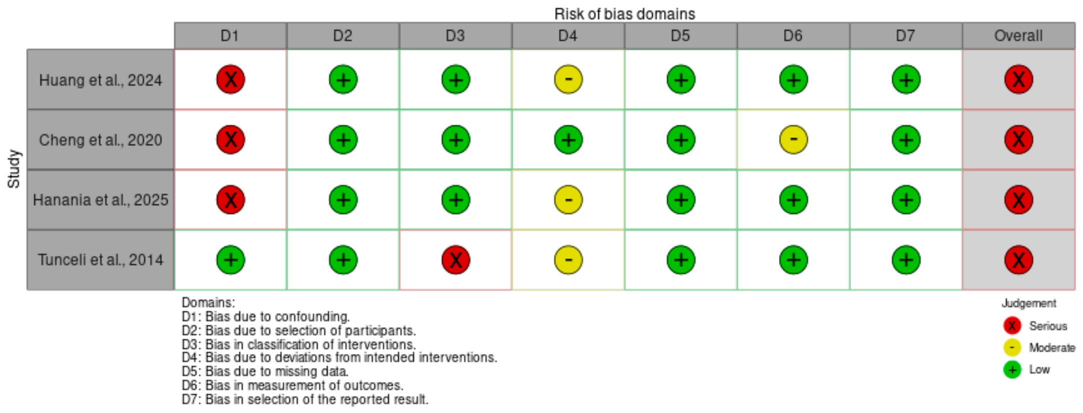

Figure S2. Risk of bias assessment for the included randomized controlled trials.

3.4. Efficacy

3.4.1. Maintenance and Reliever Therapy (MART)

Table S2. Number of patients having at least one severe exacerbation and the total number of severe exacerbation episodes in Budesonide/Formoterol as reliever and maintenance therapy.

Author, year	Endpoint	Dose of Comparison	Number of patients having ≥ 1 SE (n)	Number of SE (n)
**vs. Fluticasone/Salmeterol maintenance + Terbutaline or Sabutamol as-needed (ICS/LABA + SABA)**				
Bousquet et al., 2007 [1]	26 weeks	B/F 160/4.5 μg x 2 inh BID vs. F/S 250/50 μg BID + TEB as-needed	-	137 vs. 173
Kuna et al., 2007 [2]	26 weeks	B/F 160/4.5 μg x 1 inh BID vs. F/S 125/25 μg x 2 inh BID + TEB as-needed	94 vs. 138	125 vs. 208
Vogelmeier et al., 2005 [3]	52 weeks	B/F 160/4.5 μg x 2 inh BID vs. F/S 250/50 μg BID + SAB as-needed	159 vs. 204	-
**vs. Budesonide/ Formoterol maintenance + Terbutaline as-needed (ICS/LABA + SABA)**				
Kuna et al., 2007 [2]	26 weeks	B/F 160/4.5 μg x 1 inh BID vs. B/F 320/9 μg x 1 inh BID + TEB as-needed	94 vs. 126	125 vs. 173
O'Byrne et al., 2005 [4]	52 weeks	B/F 80/4.5 μg BID vs. B/F 80/4.5 μg BID + TEB as-needed	-	-
Atienza et al., 2013 [5]	52 weeks	B/F 160/4.5 μg x 1 inh BID vs. B/F 160/4.5 μg x 1 inh BID + TEB as-needed	170 vs. 229	259 vs. 363
Rabe et al., 2006 [6]	52 weeks	B/F 160/4.5 μg x 1 inh BID vs B/F 160/4.5 μg + TEB as-needed	143 vs. 245	194 vs. 377
Patel et al., 2013 [7]	24 weeks	B/F 200/6 μg x2 inh BID vs. B/F 200/6 μg x2 inh BID + SAB as-needed	28 vs. 50	35 vs. 66
**vs. Budesonide maintenance + Terbutaline as-needed (ICS + SABA)**				
Rabe et al., 2006 [8]	26 weeks	B/F 80/4.5 μg x 2 inh OD vs. BUD 160 μg x 2 inh OD + TEB as-needed	-	14 vs. 57
O'Byrne et al., 2005 [4]	52 weeks	B/F 80/4.5 μg BID vs. BUD 320 μg BID + TEB as-needed	-	-
Scicchitano et al., 2004 [9]	52 weeks	B/F 160/4.5 μg x 2 inh OD vs. BUD 160 μg x 2 inh BID + TEB as-needed	137 vs. 212	197 vs. 349

3.4.2. Reliever Monotherapy

Table S3. Number of patients having at least one severe exacerbation and the total number of severe exacerbation episodes in Budesonide/Formoterol as reliever therapy.

Authors, year	Endpoint	Dose of Comparison	Number of patients having ≥ 1 SE (n)	Number of SE (n)
		**Reliever: B/F as-needed vs. SABA as-needed**		
O'Byrne et al., 2018 [10]	52 weeks	PLB BID + B/F 200/6 μg as-needed vs. PLB BID + TEB 0.5 mg as-needed	71 vs. 152	77 vs. 188
Beasley et al., 2019 [11]	52 weeks	B/F 200/6 μg as-needed vs. ALB 100 μg x 2 inh as needed	-	9 vs. 23
		**Reliever: B/F as-needed vs ICS maintenance + SABA as-needed**		
Beasley et al., 2019 [11]	52 weeks	B/F 200/6 μg as-needed vs. BUD 200 μg BID + ALB 100 μg x 2 inh as-needed	-	9 vs. 21
O'Byrne et al., 2018 [10]	52 weeks	PLB BID + B/F 200/6 μg as-needed vs. BUD 200 μg BID + TEB 0.5 mg as-needed	71 vs. 78	77 vs. 89
Bateman et al., 2018 [12]	52 weeks	PLB BID + B/F 160/4.5 μg as-needed vs. BUD 200 μg BID + TEB 0.5 mg as-needed	177 vs. 184	217 vs. 221
Hardy et al., 2019 [13]	52 weeks	B/F 160/4.5 μg as-needed vs. BUD 200 μg BID + TEB 0.5 mg as needed	-	-
		**Maintenance: B/F vs. BUD**		
Lalloo et al., 2003 [14]	12 weeks	B/F 80/4.5 μg BID vs. BUD 200 μg BID	-	-
Peters et al., 2016 [15]	26 weeks	B/F 80/4.5-160/4.5 μg x2 inh BID vs. BUD 80-160 μg x2 inh BID	539 vs. 633	637 vs. 762

3.4.3. Maintenance Monotherapy

Table S4. Efficacy of B/F as maintenance therapy regarding time to first severe exacerbation, number of patients having at least one severe exacerbation, total number of severe exacerbations, and annual rate of severe exacerbation.

Authors, year	Duration	Comparison	Time to first SE		Number of patients having ≥ 1 SE (n)	Number of SE (n)	Annual SE rate		
			HR (95% CI)	*p value*			Events/patient/year	RR (95% CI)	*p value*
			**Maintenance: B/F vs. ICS**						
Lalloo et al., 2003 [14]	12 weeks	B/F 80/4.5 μg BID vs. BUD 200 μg BID	0.94	0.85	-	-	-	-	-
Peters et al., 2016 [15]	26 weeks	B/F 80/4.5-160/4.5 μg x2 inh BID vs. BUD 80-160 μg x2 inh BID	0.84 (0.74-0.94)	0.002	539 vs. 633	637 vs. 762	-	-	-

1. Bousquet, J.; Boulet, L.P.; Peters, M.J.; Magnussen, H.; Quiralte, J.; Martinez-Aguilar, N.E.; Carlsheimer, A. Budesonide/formoterol for maintenance and relief in uncontrolled asthma vs. high-dose salmeterol/fluticasone. *Respir Med* **2007**, *101*, 2437-46. doi: 10.1016/j.rmed.2007.07.014
2. Kuna, P.; Peters, M.J.; Manjra, A.I.; Jorup, C.; Naya, I.P.; Martínez-Jimenez, N.E.; Buhl, R. Effect of budesonide/formoterol maintenance and reliever therapy on asthma exacerbations. *Int J Clin Pract* **2007**, *61*, 725-36. doi: 10.1111/j.1742-1241.2007.01338.x
3. Vogelmeier, C.; D'Urzo, A.; Pauwels, R.; Merino, J.M.; Jaspal, M.; Boutet, S.; Naya, I.; Price, D. Budesonide/formoterol maintenance and reliever therapy: an effective asthma treatment option? *Eur Respir J* **2005**, *26*, 819-28. doi: 10.1183/09031936.05.00028305
4. O'Byrne, P.M.; Bisgaard, H.; Godard, P.P.; Pistolesi, M.; Palmqvist, M.; Zhu, Y.; Ekström, T.; Bateman, E.D. Budesonide/formoterol combination therapy as both maintenance and reliever medication in asthma. *Am J Respir Crit Care Med* **2005**, *171*, 129-36. doi: 10.1164/rccm.200407-884OC
5. Atienza, T.; Aquino, T.; Fernández, M.; Boonsawat, W.; Kawai, M.; Kudo, T.; Ekelund, J.; Ivanov, S.; Carlsson, L.G. Budesonide/formoterol maintenance and reliever therapy via Turbuhaler versus fixed-dose budesonide/formoterol plus terbutaline in patients with asthma: phase III study results. *Respirology* **2013**, *18*, 354-63. doi: 10.1111/resp.12009
6. Rabe, K.F.; Atienza, T.; Magyar, P.; Larsson, P.; Jorup, C.; Lalloo, U.G. Effect of budesonide in combination with formoterol for reliever therapy in asthma exacerbations: a randomised controlled, double-blind study. *Lancet* **2006**, *368*, 744-53. doi: 10.1016/s0140-6736(06)69284-2
7. Patel, M.; Pilcher, J.; Pritchard, A.; Perrin, K.; Travers, J.; et al. Efficacy and safety of maintenance and reliever combination budesonide-formoterol inhaler in patients with asthma at risk of severe exacerbations: a randomised controlled trial. *Lancet Respir Med* **2013**, *1*, 32-42. doi: 10.1016/S2213-2600(13)70007-9
8. Rabe, K.F.; Pizzichini, E.; Ställberg, B.; Romero, S.; Balanzat, A.M.; Atienza, T.; Lier, P.A.; Jorup, C. Budesonide/formoterol in a single inhaler for maintenance and relief in mild-to-moderate asthma: a randomized, double-blind trial. *Chest* **2006**, *129*, 246-256. doi: 10.1378/chest.129.2.246
9. Scicchitano, R.; Aalbers, R.; Ukena, D.; Manjra, A.; Fouquert, L.; Centanni, S.; Boulet, L.P.; Naya, I.P.; Hultquist, C. Efficacy and safety of budesonide/formoterol single inhaler therapy versus a higher dose of budesonide in moderate to severe asthma. *Curr Med Res Opin* **2004**, *20*, 1403-18. doi: 10.1185/030079904x2051
10. O'Byrne, P.M.; FitzGerald, J.M.; Bateman, E.D.; Barnes, P.J.; Zhong, N.; et al. Inhaled Combined Budesonide-Formoterol as Needed in Mild Asthma. *N Engl J Med* **2018**, *378*, 1865-1876. doi: 10.1056/NEJMoa1715274
11. Beasley, R.; Holliday, M.; Reddel, H.K.; Braithwaite, I.; Ebmeier, S.; et al. Controlled Trial of Budesonide-Formoterol as Needed for Mild Asthma. *N Engl J Med* **2019**, *380*, 2020-2030. doi: 10.1056/NEJMoa1901963
12. Bateman, E.D.; Reddel, H.K.; O'Byrne, P.M.; Barnes, P.J.; Zhong, N.; et al. As-Needed Budesonide-Formoterol versus Maintenance Budesonide in Mild Asthma. *N Engl J Med* **2018**, *378*, 1877-1887. doi: 10.1056/NEJMoa1715275
13. Hardy, J.; Baggott, C.; Fingleton, J.; Reddel, H.K.; Hancox, R.J.; et al. Budesonide-formoterol reliever therapy versus maintenance budesonide plus terbutaline reliever therapy in adults with mild to moderate asthma (PRACTICAL): a 52-week, open-label, multicentre, superiority, randomised controlled trial. *Lancet* **2019**, *394*, 919-928. doi: 10.1016/S0140-6736(19)31948-8
14. Lalloo, U.G.; Malolepszy, J.; Kozma, D.; Krofta, K.; Ankerst, J.; Johansen, B.; Thomson, N.C. Budesonide and formoterol in a single inhaler improves asthma control compared with increasing the dose of corticosteroid in adults with mild-to-moderate asthma. *Chest* **2003**, *123*, 1480-7. doi: 10.1378/chest.123.5.1480

15. Peters, S.P.; Bleecker, E.R.; Canonica, G.W.; Park, Y.B.; Ramirez, R.; Hollis, S.; Fjallbrant, H.; Jorup, C.; Martin, U.J. Serious Asthma Events with Budesonide plus Formoterol vs. Budesonide Alone. *N Engl J Med* **2016**, *375*, 850-60. doi: 10.1056/NEJMoa1511190
